# Computationally guided conversion of the specificity of E-selectin to mimic that of Siglec-8

**DOI:** 10.1073/pnas.2117743119

**Published:** 2022-10-03

**Authors:** Xiaocong Wang, Melinda S. Hanes, Richard D. Cummings, Robert J. Woods

**Affiliations:** ^a^Hubei Key Laboratory of Agricultural Bioinformatics, College of Informatics, Huazhong Agricultural University, Wuhan 430070, China;; ^b^Complex Carbohydrate Research Center, University of Georgia, Athens, GA 30602;; ^c^Department of Surgery, Beth Israel Deaconess Medical Center, Harvard Medical School, Boston, MA 02115

**Keywords:** lectin engineering, sulfated oligosaccharide, rational protein design, glycan microarray screening, GLYCAM

## Abstract

Sulfated glycans have been found to be associated with various diseases and therefore have significant potential in molecular pathology as biomarkers. Although lectins are useful reagents for detecting glycans, there is a paucity of sulfate-recognizing lectins, and those that exist, such as from *Maackia amurensis*, display mixed specificities. Recombinant lectin engineering offers an emerging tool for creating novel glycan recognition by altering and/or enhancing endogenous specificities. The present study demonstrated the use of computational approaches in the engineering of a mutated form of E-selectin that displayed highly specific recognition of 6′-sulfo-sialyl Lewis X (6′-sulfo-sLe^x^), with negligible binding to its endogenous nonsulfated ligand, sLe^x^. This new specificity mimics that of the unrelated protein Siglec-8, for which 6′-sulfo-sLe^x^ is its preferred ligand. Molecular dynamics simulations and energy calculations predicted that two point mutations (E92A/E107A) would be required to stabilize binding to the sulfated oligosaccharide with E-selectin. In addition to eliminating putative repulsions between the negatively charged side chains and the sulfate moiety, the mutations also abolished favorable interactions with the endogenous ligand. Glycan microarray screening of the recombinantly expressed proteins confirmed the predicted specificity change but also identified the introduction of unexpected affinity for the unfucosylated form of 6′-sulfo-sLe^x^ (6′-sulfo-sLacNAc). Three key requirements were demonstrated in this case for engineering specificity for sulfated oligosaccharide: 1) removal of unfavorable interactions with the 6′-sulfate, 2) introduction of favorable interactions for the sulfate, and 3) removal of favorable interactions with the endogenous ligand.

## Significance

Rational design assisted by computational approaches is a powerful and efficient tool for creating proteins specific for the recognition of novel glycans. We engineered a double mutant of E-selectin to eliminate specificity for the endogenous ligand (sLe^x^) while introducing affinity for the 6′-sulfated form of that ligand (6′-sulfo-sLe^x^). The specificity, defined by glycan array screening, identically mimics that of the unrelated protein Siglec-8, which naturally prefers to bind 6′-sulfo-sLe^x^ and its unfucosylated form (6′-sulfo-sLacNAc). We show that the rational design for this highly specific recognition for 6′-sulfation requires three core features: 1) removal of unfavorable interactions for 6′-sulfation, 2) introduction of favorable interactions for 6′-sulfation, and 3) removal of interactions that favor binding to the endogenous ligand.

Sulfation is a ubiquitous and important posttranslational modification of many biological molecules, including proteins (1), carbohydrates (2, 3), lipids (4), and glycolipids (5), and mediates many biological functions. Sulfated glycans are associated with various diseases, such as cancers (6, 7), cystic fibrosis (8–10), and osteoarthritis (11, 12), and have great potential in molecular pathology as biomarkers. However, the isolation and detection of sulfated glycans is challenging because of their low abundance in cells, their low ionization efficiency for detection by mass spectroscopy, and the fact that the modification is labile under even relatively mild isolation conditions (13, 14). Although lectins are often used to detect glycans (for example, in histology) or to enrich them chromatographically before further analysis, their application to sulfated glycans is challenging due to the paucity of sulfate-recognizing lectins as well as their broad or mixed specificities. For example, lectins from *Maackia amurensis* recognize both 3′-sulfated and 3′-sialylated oligosaccharides (15, 16), which is perhaps not that unexpected given that sulfate and sialic acid are both anionic. Even more surprising is the observation that the lectin from Langerin cross-reacts with 6′-sulfated glycans and mannose (17). Antibodies can also be used as glycan detection reagents, including antibodies with specificity for sulfated oligosaccharides (18–20).

There are also a limited number of endogenous mammalian lectins that have been found to preferentially bind to sulfated oligosaccharides, most notably members of the Siglec and selectin families (21–23). Siglecs are primarily located on the surfaces of immune cells and share a binding preference for sialylated oligosaccharides. In addition, at least 8 of the 15 known Siglecs (24) (2, 3, 5, 7, 8, 9, 14, and 15) show enhanced binding to sialylated glycans that are additionally sulfated (25, 26); however, many of these have broad specificities, with the notable exceptions of Siglec-8 and Siglec-9. Siglec-8 displays a strong preference for ligands that contain sulfation at the O6 position of galactose in sialyl Lewis X (6′-sulfo-sLe^x^; Neu5Acα2-3Gal[6S]β1-4[Fucα1-3]GlcNAcβ1) or at that position in sialyl LacNAc (6′-sulfo-sLacNAc; Neu5Acα2-3Gal[6S]β1-4GlcNAcβ1) (27). In contrast to Siglec-8, Siglec-9 prefers ligands that are sulfated at the O6 position of GlcNAc and shows enhanced glycan array binding when fucose is present; that is, 6-sulfo-sLe^x^ ≫ 6-sulfo-sLacNAc (27). These two Siglecs display orthogonal ligand specificities, and they are also selectively expressed on different leukocytes. Siglec-8 is found on eosinophils, basophils, and mast cells where it regulates their function and survival (28), while Siglec-9 is expressed by neutrophils and monocytes where it modulates the function of neutrophils during infection (29). Understanding the biological mechanisms by which subtle differences in sulfation patterns govern these specificities is an area of active research. It is worth mentioning here that the specificity of monoclonal antibody S2 parallels that of Siglec-9; although, in contrast to Siglec-9, the presence of fucose in the ligand does not enhance S2 binding (30). Thus, different proteins may display unique binding modes for the same oligosaccharide ligand.

Much of the latest data pertaining to the roles of glycan sulfation (25, 31–33) have come somewhat indirectly, and with considerable effort, from genetic studies in which activity is inferred from the impact of the transfection or deletion of sulfotransferase genes in model cell lines. Given the emerging evidence that sulfation can dramatically enhance (25, 34) or abrogate (35, 36) protein binding, the paucity of reagents that are able to detect specific sulfation patterns *in vitro* or *in vivo* creates a barrier to advancing this already challenging field. The narrow specificity of Siglec-8 presents a remarkable example of a highly specific interaction between an endogenous protein and 6′-sulfated oligosaccharides. Because such a degree of specificity is rare among naturally occurring lectins, there is a need for an alternative to serendipitous lectin discovery for the generation of novel carbohydrate detection reagents. One potential approach would be to engineer the desired specificity into an existing lectin scaffold. To be truly specific, however, the reagent should also display reduced or eliminated binding to the endogenous glycan(s). Several examples of lectin specificity engineering have been reported (37–50), with varying degrees of success.

In an early example of carbohydrate specificity engineering, based on domain swapping, Drickamer (37) introduced galactose-binding activity into a C-type lectin by substituting two amino acids that are conserved in the carbohydrate recognition domain in the mannose-specific lectin (E185 and N187) with two that are conserved in related lectins that prefer galactose. The double mutant (E185Q/N187D) indeed preferred galactose over mannose by 3.5-fold compared to the wild type, which preferred mannose over galactose by almost 14-fold. Nevertheless, the double mutant retained significant affinity for the endogenous ligands of the parent lectin and unexpectedly introduced high affinity for *N*-acetylgalactosamine. Subsequently, in the quest to develop a lectin with improved detection capability for the Thomsen–Friedenreich (TF) tumor antigen (Galβ1-3GalNAc), Adhikari et al. (38) introduced point mutations into a recombinant version of peanut agglutinin at a position (N41) that was known to stabilize a water-mediated hydrogen bond with the ligand. Replacing N41 with a glutamine enhanced affinity for the TF antigen by approximately fourfold, rationalized as arising from the replacement of the mediating water by a direct interaction with the Q41 side chain. Although the N41Q point mutation improved affinity for the target ligand, it did not narrow the endogenous specificity of the lectin. In the design of a probe for the disease marker 6-sulfo-galactose, Hu et al. (40) applied error-prone PCR to a recombinant form of the ricin B-domain lectin with endogenous affinity for galactose, work that built off their earlier success applying this approach to introduce specificity for 6′-sialyl-galactose into the same system (45). One mutation (E20K) in particular was identified as being critical for introducing sulfate binding; however, no clones were reported that significantly reduced binding to the endogenous nonsulfated ligands.

The three cases introduced above represent common approaches to lectin specificity engineering, namely, sequence-based domain swapping, structure-based point mutagenesis, and random mutation (directed evolution). Each successfully achieved the goal of introducing either novel or enhanced affinity; however, none were able to simultaneously reduce or remove affinity for the endogenous ligands. This latter property is essential to fully exploit the engineered lectin as a reagent in diagnostic or therapeutic applications.

In the present work, we sought to use computational methods to guide the design of a protein that could recognize 6′-sulfo-sLe^x^ (a ligand for Siglec-8) based on introducing sulfate specificity into a lectin (E-selectin) known to bind the nonsulfated congener. Selectins recognize the unsulfated core tetrasaccharide sLe^x^, which is found in glycoproteins, such as P-selectin glycoprotein ligand-1 (PSGL-1) (51), E-selectin-ligand-1 (52), and some CD44 isoforms (53). Sulfation of sLe^x^ can enhance, attenuate, or switch selectin specificity (21, 35, 54, 55). However, in the case of E-selectin, neither direct sulfation of sLe^x^ nor sulfation of its associated peptide enhanced its affinity (21, 56). No members of the selectin family recognize 6′-sulfo-sLe^x^ (35).

A computational approach to protein engineering has several benefits over purely experimental techniques, including the ability to predict the effect of hypothetical point mutations on ligand binding. Further, to achieve high specificity, we wanted to test the hypothesis that in addition to introducing affinity for the sulfate group, mutations could be introduced that would reduce or eliminate affinity for the endogenous nonsulfated oligosaccharide. E-selectin was chosen to demonstrate this approach as its specificity and three-dimensional (3D) structure have been reported previously, and it has been shown to have no measurable affinity for 6′-sulfo-sLe^x^ (35). The results from this study may offer insight into the rules governing oligosaccharide specificity and provide a rational and generalizable approach to developing carbohydrate-specific reagents.

## Results

### Molecular Models for E-Selectin and Siglec-8 Complexes.

To confirm the validity of the molecular modeling protocol, molecular dynamics (MD) simulations (200 ns each) were first performed on complexes of E-selectin and Siglec-8 with their cognate ligands sLe^x^ and 6′-sulfo-sLe^x^, respectively, as reported from experimental structural studies (57, 58). Additionally, to permit a statistical assessment of the variability in the data, three independent MD simulations were performed for each complex. The MD simulation data reproduced the experimentally observed ligand binding poses and glycosidic linkage values (*SI Appendix*, Fig. S1) and the interatomic interactions (57, 58) (Table 1 and *SI Appendix*, Table S1). In the case of E-selectin, the endogenous sLe^x^ ligand maintained stable hydrogen-bond interactions with the protein via the sialic acid, galactose, and fucose residues, as observed in the crystal structure (57). In the case of Siglec-8, the endogenous 6′-sulfo-sLe^x^ ligand also maintained the key experimentally observed interactions during the simulations (Table 1 and *SI Appendix*, Table S1), particularly with R56, R109, and K116, residues that are known to be critical for affinity based on experimental alanine scanning (58). Notably, MD simulations of the R56A, R109A, and K116A mutants (computational alanine scanning) in the complex with 6′-sulfo-sLe^x^ showed that, consistent with the experimental affinity data (58), the loss of R109 completely abolished affinity for the 6′-sulfo-sLe^x^ ligand, as evidenced by the ligand diffusing out of the binding site (*SI Appendix*, Fig. S2). Data from molecular mechanical generalized Born surface area (MM-GBSA) binding energy analyses (59) also supported the experimental observations that the R56A and K116A mutations weaken affinity but do not abolish it (*SI Appendix*, Fig. S2 and Table S2).

**Table 1.** Stable intermolecular hydrogen-bond pairs observed in the MD simulations for E-selectin and Siglec-8 complexes with their endogenous ligands and the E92A/E107A–6′-sulfo-sLe^x^ complex

		E-selectin–sLe^x^	E92A/E107A–6′-sulfo-sLe^x^	Siglec-8–6′-sulfo-sLe^x^
Neu5Ac	CO_2_^−^	Y48*, R97*	Y48, R97	R109*
	O4	E98	E98	—
	N5	—^†^	—	K116*
	O7	—	—	Y7*
	O8	—	—	Y58, R109
Core-2 Gal	O3	R97	R97	—
	O4	Y94, R97	Y94, R97	—
	O6	E92*	—	—
Fuc	O2	E88*	E88	—
	O3	E88*, Ca^2+^	E88, Ca^2+^	—
	O4	E80*, N82*, Ca^2+^	E80, N82, Ca^2+^	—
SO_3_ (6′)	O	—	N105, K111, K113	R56*, Q59*

^*^Observed in experimental structures.

^†^No stable interactions observed.

Having thus confirmed the ability of the molecular modeling protocols to reproduce the experimentally observed conformations and interactions for the known E-selectin–sLe^x^ and Siglec-8–6′-sulfo-sLe^x^ complexes, we applied the computational mutagenesis method to design 6′-sulfation recognition into E-selectin.

### Engineering 6′-Sulfation Recognition into E-Selectin.

**Fig. 1.**
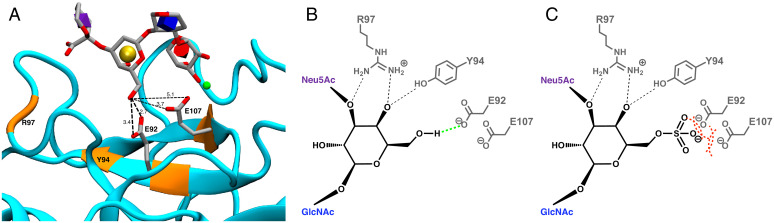
(*A*) The crystal structure (PDB ID: 4CSY [57]) of sLe^x^ in the binding site of E-selectin (cyan), with monosaccharides shown in licorice representation and their identities shown with 3D-Symbol Nomenclature for Glycans (SNFG) nomenclature (fucose, red cone; GlcNAc, blue cube; galactose, yellow sphere; Neu5Ac, purple diamond) inside each ring (60). The requisite Ca^2+^ ion is shown in green. Key amino acid positions for galactose binding (orange) include E92 and E107, for which the side chains and interatomic distances (Å) between the carboxylate oxygen atoms and Gal-O6 are shown. (*B*) Schematic representation of the hydrogen bonds (dashed lines) involving the galactose residue in the wild-type E-selectin–sLe^x^ complex, showing (in green) the key stabilizing interaction between E92 and the Gal-O6 position (Table 1 and *SI Appendix*, Table S1). (*C*) Schematic representation of the putative unfavorable interactions involving the sulfate moiety (dashed red curves).

To initiate the engineering of E-selectin to recognize 6′-sulfo-sLe^x^, MD simulations of E-selectin in complex with the target ligand (6′-sulfo-sLe^x^) were performed, in which the sulfated ligand was generated by replacing the 6-OH with a sulfate moiety (*Materials and Methods*). Consistent with the experimental observation that E-selectin does not show measurable affinity for this sulfated ligand (35), the complex was unstable during each of the three independent simulations (*SI Appendix*, Fig. S3). A closer examination of the trajectories showed that ligand instability was accompanied by distortions of the glycosidic linkages into high-energy conformations (*SI Appendix*, Fig. S4). Instability of the complex precluded calculation of the interaction energy for this system; however, examination of the E-selectin–6′-sulfo-sLe^x^ complex suggested that the ligand instability likely arose from the presence of unfavorable van der Waals and electrostatic interactions between the sulfate moiety and the side chains of glutamate residues E92 and E107. In the E-selectin–sLe^x^ crystal structure, the 6-OH group in the galactose residue is in close proximity to the side chains of E92 and E107, with a hydrogen bond present between it and the E92 carboxylate group (Fig. 1). Consequently, sulfation of the O6-group would result in unfavorable van der Waals and electrostatic interactions with the side chains of at least one of the glutamate residues. To fully investigate the impact of each of the negatively charged side chains in E92 and E107, ligand complexes with two single mutations (E92A and E107A) and a double mutation (E92A/E107A) were computationally analyzed, with the expectation that the smaller uncharged side chain of alanine would remove the unfavorable interactions with the sulfate moiety.

### E92A in E-selectin–6′-sulfo-sLe^x^.

**Fig. 2.**
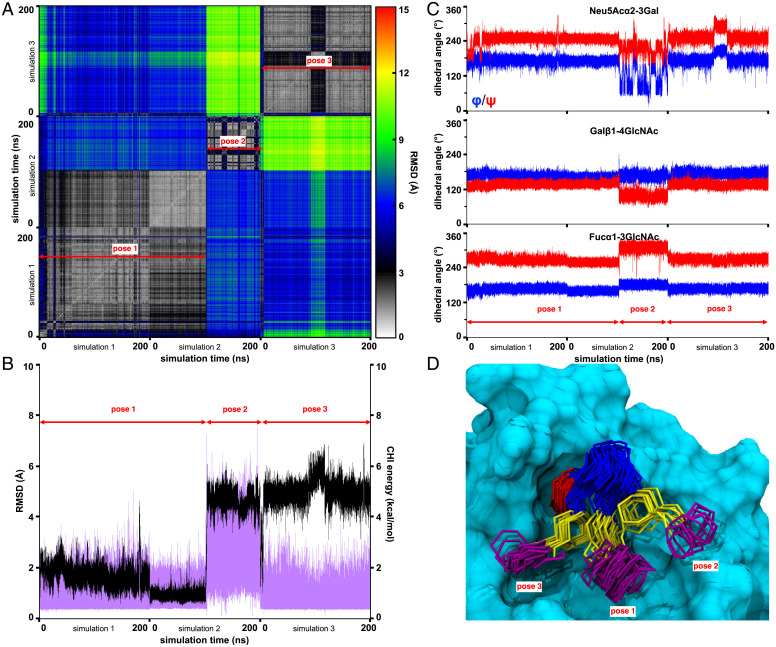
Analysis of the stability of 6′-sulfo-sLe^x^ in the binding site of the mutant E92A. (*A*) Two-dimensional positional RMSD plot for the ring atoms in the ligand. Structures were evenly extracted every 0.1 ns from three MD simulations, and structurally related poses for the ligand are labeled (*Bottom*). (*B*) Trajectories of the positional RMSD (black) for the ring atoms and the total CHI energy (61) for the glycosidic linkages (purple) in the ligand in three MD simulations. The RMSD values were computed relative to the position of the endogenous ligand in the crystal structure of the E-selectin–sLe^x^ complex (57). (*C*) Glycosidic linkages trajectories (φ: blue; ψ: red) for the ligand in three independent MD simulations; φ = C2-C1-Ox-Cx (for Neu5Ac: φ = C3-C2-Ox-Cx) and ψ = C1-Ox-Cx-Cx-1 (for Neu5Ac: ψ = C2-Ox-Cx-Cx-1). (*D*) Superimposition of the complex for MD frames evenly extracted every 10 ns showing only the pyranose ring and glycosidic linkage atoms for clarity. The monosaccharides are colored according to SNFG nomenclature (62, 63), and the protein solvent-accessible surface is shown in cyan.

The E92A mutation abolishes the hydrogen bond with the 6-OH moiety in galactose observed in the wild-type E-selectin–sLe^x^ complex and concurrently eliminates putative repulsions between the sulfate moiety in 6′-sulfo-sLe^x^. This mutation is therefore potentially important for both decreasing the affinity of the endogenous ligand and enhancing that of the target ligand. Indeed, when complexed with E92A, the 6′-sulfo-sLe^x^ ligand remained bound, although disordered, throughout each independent MD simulation, in contrast to the high degree of instability observed in the complex with wild-type E-selectin. Nevertheless, the ligand populated three distinct poses (pose1, pose2, and pose3 in Fig. 2), indicative of a high degree of disorder and instability. In the complex with E92A, pose1 and pose3 adopted a similar ligand shape (Fig. 2 and *SI Appendix*, Fig. S3), which is equivalent to that seen in the experimental co-complex with its cognate receptor Siglec-8 (58), but each pose adopted different orientations relative to the mutant protein surface (Fig. 2 and *SI Appendix*, Table S3). By contrast, the unique shape of the ligand in pose2 resulted from an unexpected flip of the GlcNAc ring from ^4^C_1_ to ^1^C_4_, likely induced by the initial placement of the ligand in the hypothetical E92A binding site. Thus, this single point mutation was predicted to be insufficient to lead to a complete conversion in ligand specificities.

To quantify these predictions, the interaction energies for E92A with the 6′-sulfo-sLe^x^ and with the nonsulfated ligand were computed. Pose1 and pose2 both displayed unfavorable binding interaction energies, 0.4 and 5.0 kcal/mol, respectively (*SI Appendix*, Table S4). Although pose3 showed a strong favorable binding interaction energy (−7.6 kcal/mol; *SI Appendix*, Table S4), the average binding interaction energy from the three independent MD trajectories (−0.7 kcal/mol) indicated a significantly weaker affinity than the wild-type E-selectin–sLe^x^ complex (−3.9 ± 1.0 kcal/mol; Table 2). Without the binding contribution from the 6′-sulfo moiety, the complex of E92A–sLe^x^ displayed a negligible binding interaction (−0.2 ± 2.1 kcal/mol; *SI Appendix*, Table S4), despite the observation that the ligand maintained positional stability in the binding site (*SI Appendix*, Fig. S5). In the present energy calculations, we applied a value of 3.0 for the internal dielectric constant of the proteins in the MM-GBSA analysis. It should be noted that values greater than 1 for the internal dielectric constant are proposed to serve as an approximate method to account for the effect of charge polarization induced by ligand binding and are therefore somewhat system dependent, with values of 2 to 4 being proposed (64, 65). In the present case, the interaction energy computed with an internal dielectric constant of 3.0 (−3.9 ± 1.0 kcal/mol) agrees well with the known binding affinity (−4.2 kcal/mol) (66) for the E-selectin–sLe^x^ complex. Interestingly, an internal dielectric constant of 3.0 also reproduced the interaction energy for Siglec-8 with its cognate ligand 6′-sulfo-sLe^x^ reasonably well (theoretical binding energy of −5.1 ± 0.6 kcal/mol [Table 2] and experimental affinity of −4.8 kcal/mol [58]).

**Table 2.** Per-residue interaction energies* and entropic penalties^†^ for wild-type and mutated E-selectins and Siglec-8 complexes with their ligands.

	E-selectin–sLe^x^	E92A/E107A–6′-sulfo-sLe^x^	Siglec-8–6′-sulfo-sLe^x^
**Per-residue interaction energies (MM-GBSA) and percentage of binding**			
Neu5Ac	−3.6 ± 0.1 (14%)	−3.2 ± 0.6 (11%)	−12.6 ± 0.2 (64%)
Core-2 Gal	−5.8 ± 0.5 (23%)	−4.7 ± 0.4 (17%)	−3.3 ± 0.0 (17%)
GlcNAc	−2.8 ± 0.1 (11%)	−3.4 ± 0.1 (12%)	−2.5 ± 0.3 (12%)
Fuc	−13.2 ± 0.4 (52%)	−13.5 ± 0.0 (48%)	0.0 ± 0.0 (<1%)
SO_3_ (6′)	—	−3.3 ± 0.6 (12%)	−1.3 ± 0.2 (7%)
Δ*G*_MM/GBSA_	−25.4 ± 0.7	−28.1 ± 1.6	−19.7 ± 0.3
**Entropic penalties**			
−TΔ*S*_RTV (all)_	19.2 ± 1.1	19.7 ± 0.9	13.3 ± 0.6
−TΔ*S*_q_^C^	2.3 ± 0.0	2.4 ± 0.2	1.3 ± 0.1
−TΔ*S*	21.5 ± 1.1	22.1 ± 1.1	14.6 ± 0.5
**Binding free energies**			
Δ*G*_binding_	−3.9 ± 1.0	−6.0 ± 0.8	−5.1 ± 0.6

^*^In kcal/mol.

^†^At 300 K.

### E107A in E-Selectin–6′-sulfo-sLe^x^.

In the E-selectin–sLe^x^ crystal structure (Fig. 1), the negatively charged carboxylate group in the side chain of E107 forms only a very weak interaction with the O6 hydroxyl group in galactose (3.7 Å), in contrast to the strong hydrogen bond formed between that hydroxyl group and the carboxylate in E92. Consequently, we would infer that were a sulfate group present at O6, both the carboxylate groups in E92 and E107 would be repulsive, with the former being more so. The MD simulations confirmed that the E107A complex was only marginally stable (*SI Appendix*, Fig. S6), presumably due to repulsions from interactions with E92. Over the three independent MD simulations of the E107A complex, the sulfated ligand adopted approximately four poses (*SI Appendix*, Fig. S7), with the oligosaccharide pose equivalent to that seen in the wild-type E-selectin–sLe^x^ complex being present for only ∼20% of the simulation. The three other poses displayed large positional and conformational variations, in which the glycosidic linkages adopted higher energy orientations than those in the stable E-selectin–sLe^x^ complex. Due to the instability of the E107A complex in each of the three independent 200-ns simulations, the entropy values failed to converge. For this reason, the energy values were not computed.

Collectively, each of the single mutations of E-selectin only partially stabilized the binding of 6′-sulfo-sLe^x^ to E-selectin, and it appeared that mutating only one glutamate (E92A or E107A) was insufficient to fully eliminate the unfavorable electrostatic and van der Waals interactions with the 6′-sulfate moiety in the ligand. While each single point mutation weakened binding to the endogenous ligand (sLe^x^), the simulations showed that the ligand remained bound, if also highly disordered. With an internal dielectric value of 3.0, the E92A mutation weakened binding to sLe^x^ by 3.7 kcal/mol, nearly abolishing it, and for the E107A mutation, the affinity was reduced by 2.9 kcal/mol (*SI Appendix*, Tables S4 and S5).

### E92A/E107A in E-Selectin–6′-sulfo-sLe^x^.

Unlike the wild-type E-selectin and its two single mutants, the complex for the double mutant with 6′-sulfo-sLe^x^ was stable during each of the three independent MD simulations, with the ligand adopting the same single binding pose as seen in the endogenous sLe^x^ ligand bound to wild-type E-selectin (*SI Appendix*, Figs. S6 and S8). The stability of this complex indicated that the E92A/E107A double mutation significantly reduced any unfavorable electrostatic or van der Waals interactions with the 6′-sulfate moiety. The stability of the ligand correlated with the presence of strong hydrogen bonds between the ligand and the protein, particularly those involving the sialic acid (carboxylate group and 4-OH), fucose (2-OH, 3-OH, and 4-OH), and galactose (3-OH and 4-OH) residues (Table 1). While the double mutation caused the loss of a hydrogen bond between the 6-OH group in galactose and the side chain of E92, new interactions were formed between the 6′-sulfo group and the side chains of polar residue N105 and positively charged residues K111 and K113 (Table 1).

The binding energies for the E92A/E107A–6′-sulfo-sLe^x^ complex were computed to permit quantitative comparison with the wild-type E-selectin–sLe^x^ complex. The per-residue MM-GBSA energy analysis of the E92A/E107A–6′-sulfo-sLe^x^ complex showed that the fucose residue contributed more to the total binding energy (48%) than did the sialic acid residue (11%; Table 2). This energy distribution was similar to that seen in the E-selectin–sLe^x^ complex (fucose, 52%; sialic acid, 14%; Table 2). These MM-GBSA analyses are consistent with the experimental observation that removing the fucose residue from the sLe^x^ glycan in the PSGL-1 glycopeptide ligand reduced binding below the detection limit (65, 67). The binding energy for the oligosaccharide component (that is, the ligand not including the sulfate group) in the E92A/E107A–6′-sulfo-sLe^x^ complex (−24.8 ± 1.0 kcal/mol) appeared to be slightly weaker than the endogenous sLe^x^ in the wild-type E-selectin complex (−25.4 ± 0.7 kcal/mol); however, this difference is not statistically significant (*P* = 0.4425). Thus, the enhanced binding of the sulfated ligand (−2.7 kcal/mol, not including entropy) can be attributed predominantly to new interactions formed with the sulfate moiety (−3.3 ± 0.6 kcal/mol).

The MM-GBSA analysis quantifies the magnitude of the energy gained from the formation of hydrogen-bond interactions between the sulfo group and N105, K111, and K113 and that lost with the abolition of the hydrogen bond between the 6-OH group in galactose and the side chain of E92. The impact of the double mutations on the binding affinity of the endogenous sLe^x^ ligand was determined from MD simulations of the putative complex of E92A/E107A with sLe^x^. This complex was stable in each of the three independent MD simulations (*SI Appendix*, Fig. S5) and gave rise to a binding energy of −24.1 ± 0.5 kcal/mol, not including entropy. Although stable, the interaction energy was reduced by ∼4 kcal/mol compared to that of the E92A/E107A–6′-sulfo-sLe^x^ (*SI Appendix*, Table S5). With the inclusion of entropic effects, the absolute binding energy of the E92A/E107A–sLe^x^ complex was −2.4 ± 0.9 kcal/mol compared to −6.0 ± 0.8 kcal/mol for the binding of the 6′-sulfo-sLe^x^ ligand.

The computational analysis predicted that to engineer recognition of 6′ sulfation into E-selectin would require removing unfavorable electrostatic and van der Waals interactions between the 6′-sulfo group and the negatively charged side chains of E92 and E107. The analysis also predicted that replacing only one of these side chains would only partially stabilize the 6′-sulfo-sLe^x^ ligand in the binding site. To fully stabilize the complex, a double mutation (E92A and E107A) was required. In the double mutant, the 6′-sulfo-sLe^x^ oligosaccharide bound in the same low-energy pose observed in the endogenous nonsulfated ligand and formed additional strong interactions involving the sulfate group. Moreover, specificity for the novel sulfated ligand over the endogenous oligosaccharide was predicted to arise from loss of a hydrogen bond to 6-OH in galactose after mutation of E92. Having obtained statistically robust data from MD simulations and MM-GBSA analyses, we then undertook the expression of the relevant mutants of E-selectin in HEK293 cells with the aim of experimentally confirming their specificity by glycan array screening.

### Glycan Microarray Data for Wild-Type and Mutated E-Selectins.

**Fig. 3.**
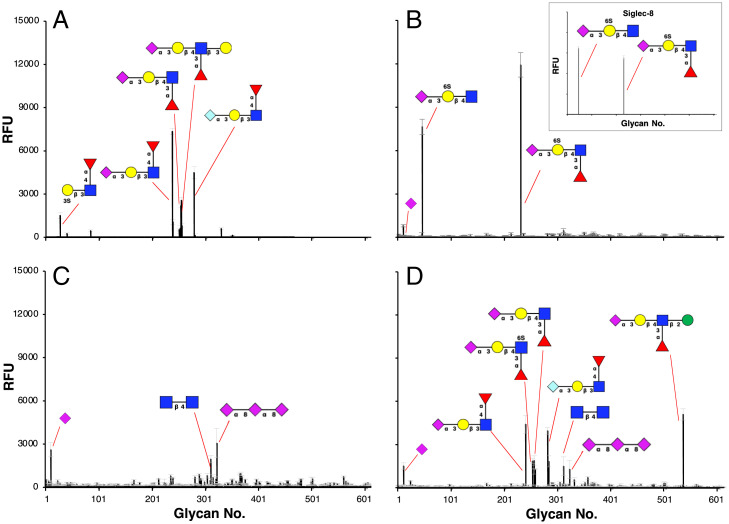
Reported glycan microarray data for the wild-type E-selectin (64) (*A*). Glycan microarray data measured at the NCFG for E92A/E107A (*B*), E92A (*C*), and E107A (*D*). Reported glycan microarray data for Siglec-8 (69) are shown in the inset of *B*; RFU, relative fluorescence units.

The recombinant E-selectin mutants were submitted to the National Center for Functional Glycomics (NCFG) for glycan microarray screening. Glycan array data for wild-type human E-selectin have been previously reported (68) and, as expected, showed binding to a limited number of sialic acid–containing glycans, including sLe^a^ and sLe^x^ (Fig. 3*A*). By contrast, the E92A/E107A double mutant displayed exclusive specificity for the 6′-sulfo sialylated lactosamine (6′-sulfo-sLacNAc) motif present in 6′-sulfo-sLe^x^ (Fig. 3*B*), a specificity indistinguishable from that of the wild-type Siglec-8. Neither single mutation alone was sufficient to generate a binding signal to 6′-sulfo-sLe^x^ (Fig. 3 *C* and *D*). The loss of detectable affinity for the endogenous sLe^x^ in the double mutant is consistent with the modeling-based interpretation that the double mutation not only enhanced the binding to 6′-sulfo-sLe^x^ by introducing new interactions with 6′-sulfo group but also reduced the affinity for the endogenous sLe^x^ ligand by removing a key hydrogen bond between the Gal-O6 hydroxyl group and E92. Therefore, the specificity of the double mutant demonstrates the importance of combining mutations that enhance binding to the target ligand with ones that attenuate binding to the endogenous ligand. That the computed binding energy for the E92A/E107A–sLe^x^ complex (Δ*G*_binding_ = −2.4 ± 0.9 kcal/mol) was ∼3.6 kcal/mol weaker than the binding of the 6′-sulfo-sLe^x^ ligand suggested that the double mutant retained some affinity for the nonsulfated ligand. However, the fact that this interaction was not observed by glycan array screening indicated that any remaining affinity must be below the detection limit of the experimental assay.

The observation that the double mutant bound to 6′-sulfo-sLacNAc was unexpected given that E-selectin–ligand interactions are characterized by a coordination between the O3 and O4 hydroxyl groups of the fucose residue and the Ca^2+^ ion, leading to the classification of this protein as a C-type lectin (70). To confirm the requirement for Ca^2+^ in wild-type E-selectin binding and to define this dependence for the recombinant mutants, the glycan array screening experiments were repeated for each system in the presence of 10 mM ethylenediaminetetraacetic acid (EDTA). Under these conditions, no binding to any ligands was observed (*SI Appendix*, Fig. S9). This result would be expected for binding that depends on coordination of the fucose ring to the Ca^2+^ ion but suggested that some alternative Ca^2+^-dependent mode of interaction must be present to explain loss of binding of 6′-sulfo-sLacNAc in the presence of EDTA.

To establish a molecular mechanism for the binding of 6′-sulfo-sLacNAc to the double mutant, an initial model for this complex was generated based on the structure of this mutant bound to 6′-sulfo-sLe^x^. The fucose was then removed, and the complex was subjected to three independent MD simulations (200 ns). During the MD simulations, the ligand remained bound to the mutant but adopted a modified orientation (ring atom RMSDd of ∼4 Å) compared to the initial ligand position (*SI Appendix*, Figs. S6 and S10), arising from a change in the conformation of the Neu5Acα2-3Gal glycosidic linkage angles (*SI Appendix*, Fig. S11). In the absence of the fucose residue, the reorientated ligand retained its characteristic interactions between the sialic acid and the protein (*SI Appendix*, Table S6) but was now able to form a stable water-mediated interaction between the sulfate moiety and the Ca^2+^ ion. This interaction was detected by performing an analysis of high-occupancy water positions in the MD trajectory and was confirmed to be present in all three independent MD simulations (distance for Ca^2+^–O (H_2_O) of 2.0 ± 0.1 Å; distance for O (H_2_O)–O (SO_3_) of 3.0 ± 0.8 Å; *SI Appendix*, Fig. S12). For reference, a similar water-mediated interaction between a sulfate group and a Ca^2+^ ion has been reported in human annexin V [Protein Data Bank ID [PDBID] 1AVR (71)] with a Ca^2+^–O (H_2_O) distance of 2.4 Å and a O (H_2_O)–O (SO_3_) distance of 2.9 Å). The presence of a persistent interaction between the sulfate moiety and the Ca^2+^ ion, albeit water mediated, provides a rationalization for the observed Ca^2+^-dependent nature of the binding between 6′-sulfo-sLacNAc and the double mutant and simultaneously explains the abolition of a requirement for fucose in the ligand. An equivalent interaction was not observed in the complex of the double mutant with 6′-sulfo-sLe^x^.

A closer examination of the MD data indicated that the position of this mediating water molecule corresponds to that of one of the Ca^2+^-coordinated water molecules in the apo–E-selectin crystal structure [water molecule 315 in PDBID 1ESL (72)] and also to the position that is occupied by fucose hydroxyl group O3 after binding to sLe^x^ in the reported crystal structure (PDBID 4CSY). It thus appears that the double mutant displays two modes of ligand recognition, one that parallels the canonical C-type lectin binding of E-selectin with additional interactions between the sulfate moiety and residues N105, K111, and K113 and an alternative mode that may occur in the absence of fucose, wherein the sialic acid and sulfate moieties maintain their key interactions with the protein but in which the sulfo group additionally forms a water-mediated electrostatic interaction with the Ca^2+^ ion (*SI Appendix*, Table S6). This fascinating possibility will be the subject of further theoretical and experimental investigation.

### Orthogonal Binding Modes Display Equivalent Ligand Specificity.

**Fig. 4.**
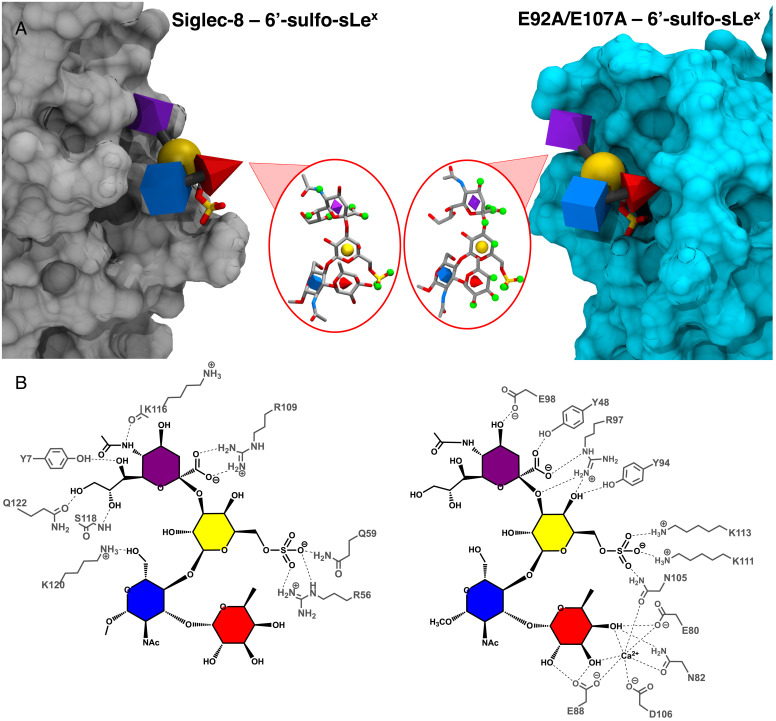
(*A*) Representative structures for Siglec-8–6′-sulfo-sLe^x^ and E92A/E107A–6′-sulfo-sLe^x^ complexes from their MD simulations. Atoms involved in hydrogen bonds with the protein surfaces (Table 1) are shown as small green spheres. Monosaccharides are drawn and colored according to 3D-SNFG nomenclature (60) (fucose, red cone; GlcNAc, blue cube; galactose, yellow sphere; Neu5Ac, purple diamond); 6′-sulfo groups are shown in stick model. Protein solvent-accessible surfaces were computed with VMD (74), with Siglec-8 shown in gray and E92A/E107A shown in cyan. Note, the viewing angle is different from that presented in Fig. 2. (*B*) Schematic representations of binding sites showing hydrogen bonds as dashed lines.

The selectivity displayed in the glycan microarray for E92A/E107A appeared to be indistinguishable from that reported for wild-type Siglec-8 (28, 73) (Fig. 3*B*), suggesting that the recognition of 6′-sulfo-sLe^x^ has been engineered into E-selectin by the double mutation. Examination of the conformations for 6′-sulfo-sLe^x^ in the MD simulations of the complexes with E92A/E107A and Siglec-8 showed that the glycosidic linkages of the ligand in both complexes displayed the same distributions (*SI Appendix*, Figs. S1 and S6), indicating that the 6′-sulfo-sLe^x^ ligand adopted the same conformation in each complex (Fig. 4). Remarkably, the engineered 6′-sulfo-sLe^x^ recognition motif in the E92A/E107A is not equivalent to that in the wild-type Siglec-8 (Fig. 4). Whereas the binding of 6′-sulfo-sLe^x^ to the E-selectin double mutant is driven predominantly by interactions with the sulfate moiety, the sialic acid, and the fucose (via coordination to a Ca^2+^ ion conserved across the selectins), in the complex with Siglec-8, the affinity arose primarily from interactions with the sulfate moiety and the sialic acid (Table 2). Similar to the wild-type E-selectin–sLe^x^ complex, in the double mutant, the fucose contributed nearly half of the total binding energy from 6′-sulfo-sLe^x^. By contrast, in the wild-type Siglec-8–6′-sulfo-sLe^x^ complex, the fucose made a negligible contribution to binding, while the sialic acid residue contributed more than half of the total binding energy. The sulfate group in the Siglec-8–6′-sulfo-sLe^x^ complex was predicted to contribute −1.3 ± 0.2 kcal/mol to the total binding energy, which may be compared to a value of −3.3 ± 0.6 kcal/mol for the interaction with the same moiety in the E92A/E107A mutant. The higher magnitude of the binding energy attributable to the sulfate in the double mutant complex is consistent with the observation that in the double mutant, the sulfate interacts with two charged residues (K111 and K113), while in the complex with Siglec-8, it interacts only with one charged residue (R56). Overall, the energy decomposition pattern correlated well with the presence of hydrogen bonds observed in the MD simulations (Table 1 and *SI Appendix*, Table S1).

## Discussion

The present modeling study demonstrated the use of MD simulations and per-residue energy analyses to rationally guide the engineering of sulfate-binding specificity into a nonsulfate-binding protein. Specifically, the modeling was used to convert the specificity of E-selectin from a preference for its endogenous ligand (sLe^x^) to the 6′-sulfated form of the same oligosaccharide (6′-sulfo-sLe^x^). MD simulations predicted that E-selectin does not recognize 6′-sulfo-sLe^x^ due to repulsions between the negatively charged side chains of E92 and E107 and the 6′-sulfo group. Additionally, simulations suggested that removing only one of the negatively charged side chains was not sufficient to stabilize binding of the sulfated ligand. However, a simultaneous double mutation, which eliminated the unfavorable repulsions by removing negatively charged side chains, reduced affinity to the endogenous sLe^x^ ligand with a loss of a key hydrogen bond and introduced new favorable interactions to the 6′-sulfo group. These predictions were confirmed experimentally by screening the double mutant (expressed in HEK293 cells) against a glycan array containing more than 600 glycans, which confirmed not only that the double mutant exclusively bound to glycans terminating in the 6′-sulfo-sialyl motif but also that neither the wild-type E-selectin nor either of the single mutants showed this specificity.

The present study demonstrated a successful example of the rational design of a protein-based probe for a sulfated glycan through modeling the effects of site-directed mutagenesis. The work also led to the serendipitous discovery of a putative interaction mode for nonfucosylated 6′-sulfo-sLe^x^. As predicted computationally and confirmed experimentally, a double mutation was required to introduce the desired specificity. Notably, the computational analyses indicated that engineering the desired specificity required three components:

1) Removal of destabilizing steric and electrostatic interactions between the 6′-sulfate and E92 and E107.
2) Creation of favorable electrostatic interactions between the 6′-sulfo group and K111, K113, and N105, enabled by the E92A/E107A mutations.
3) Loss of a favorable hydrogen bond from Gal-O6 in the endogenous ligand to E92.

The first two components enhanced affinity for the novel ligand, while the third was required to eliminate affinity for the endogenous glycan. Indeed, it seems reasonable that introducing specificity required not only the creation of favorable interactions with the new ligand but also the introduction of interactions that disfavored binding to the endogenous ligand.

## Materials and Methods

### Structure Preparation.

The initial coordinates for E-selectin–sLe^x^ and Siglec-8–6′-sulfo-sLe^x^ complexes were obtained from the PDB (entry codes 4CSY [57] and 2N7B [58], respectively). Chain A was extracted from the E-selectin–sLe^x^ complex crystal structure, with the water molecules removed. Mutants of Siglec-8 (R109A, R56A, and K116A) and E-selectin (E92A, E107A, and E92A/E107A) were created by removing the extra atoms in the side chain of the corresponding residues. Both the addition of sulfate group to the O6 position of the galactose residue in sLe^x^ and the removal of the fucose residue from sLe^x^ in the E-selectin complexes were performed by using the tLEaP module in AMBER15 (75). Force field parameters for sLe^x^ and the 6′-sulfo group were taken from the GLYCAM06 (version j) (76) parameter set, and those for the proteins were taken from AMBER15 (ff99sb) (77). Sodium or chloride counter ions were added to neutralize each protein complex using the tLEaP module before they were solvated in a truncated octahedral box (8-Å buffer with transferable intermolecular potential 3-point [TIP3P] water model).

### Simulation Setup.

Energy minimizations of the solvated complexes were performed under canonical ensemble (nVT) conditions with a two-step procedure. First, the positions of water molecules and counter ions were restrained (100 kcal/mol·Å^2^). Second, all restraints were removed except for Cα atoms on the protein backbone and ring atoms in glycan, and the minimization cycle was repeated. After energy minimization, each system was heated to 300 K over 50 ps (nVT) with a restraint (10 kcal/mol·Å^2^) on the same atoms as those in the previous step. Before data collection, systems were equilibrated at 300 K under the isothermal-isobaric ensemble (nPT) with a Berendsen thermostat (78) for 10 and 0.5 ns, consecutively. In the first equilibration, the same restraint as that in the heating process was applied. Then, the restraints on the ring atoms in glycan were removed in the second equilibration.

Production MD simulation for each complex was performed for 200 ns with the graphics processing unit (GPU) implementation of PMEMD from the AMBER15 software package (79) with the same restraints in the previous step. In all MD simulations, covalent bonds involving hydrogen atoms were constrained using the SHAKE algorithm (80), allowing a simulation time step of 2 fs. A nonbonded cutoff of 8 Å was applied to van der Waals interactions, with long-range electrostatics treated with the particle mesh Ewald approximation. Standard 1–4 nonbonded scale factors (1.0 and 2.0/1.2) were applied within the ligand and protein, respectively (76). MD simulations for all complexes were performed independently three times.

### Binding Free Energy, Entropy, Carbohydrate Intrinsic (CHI) Energy Calculations, and Representative Ligand Structure Extraction.

The MM-GBSA calculations for binding interaction energies and per-residue contributions were performed on 5,000 snapshots extracted evenly from 200 ns of MD simulation using a single-trajectory method with the MMPBSA.py.MPI module in AMBER. The GB_1_^OBC^ model (81) and internal dielectric constant (*ε*_int_) of 3.0 were applied in all MM-GBSA calculations. Quasiharmonic (QH) entropies (Δ*S*_RTV_) were calculated using the cpptraj module in AMBERTOOLS (82) and fit linearly as a function of inverse simulation period. The intercept with the *y* axis of the linear fitting function is the extrapolation of QH entropy to an infinite simulation period (83). Conformational entropies associated with changes in the glycosidic torsion angle distributions that occur after binding were computed using the Karplus–Kushick approach (84). CHI energies associated with the glycosidic linkages in the ligand were computed with the corresponding torsion angles from the MD simulation trajectories and the reported energy curves (61). The CHI energies for the glycosidic linkages in Neu5Acα2-3Gal were not included. The CHI energies for the glycosidic linkages in Fucα1-3GlcNAc were computed by using the mirror image of the reported energy curves (61). Conformation of the ligand that was most similar to its average shape in the protein complex acquired from all three independent MD simulations was extracted and presented as its representative structure. The analyses of high-occupancy water positions in the MD simulation trajectories were performed with the visual molecular dynamics (VMD) volmap plugin (74), which computed the average densities of water molecules over all matrices of cubic voxels (a cell size of 0.5 Å).

### Cloning and Protein Expression.

The gene for human E-selectin (including residues 22 to 558) was designed to include the transferrin secretion signal and C-terminal human Fc and 8×His tag and purchased from Genewiz. The resulting gene was cloned into pcDNA3.1 for expression in mammalian cells. The wild-type construct was used as a template to create the mutants E92A and E107A and the double mutant E92A/E107A using Quickchange mutagenesis. HEK293 Freestyle cells were transiently transfected with the expression construct using polyethylenimine, and culture supernatants were harvested 5 to 7 d after transfection. E-selectins were purified from the culture supernatants by nickel affinity chromatography and dialyzed against storage buffer (20 mM Tris-HCl, 250 mM NaCl, and 5 mM CaCl_2_, pH 7.4) and flash frozen and stored at −80 °C until use.

### Microarray Experiments.

The E-selectin proteins were run on consortium for functional glycomics (CFG) version 5.2 microarrays (85, 86). Microarray slides were rehydrated for 5 min in TSM buffer (20 mM Tris-HCl, 150 mM NaCl, 2 mM CaCl_2_, and 2 mM MgCl_2_) before adding 50 μg/mL of Fc-tagged human E-selectins in TSM binding buffer (TSM buffer with 1% bovine serum albumin). Microarrays were washed with TSM + 0.05% Tween-20, and bound selectins were detected with anti-human IgG-Alexa488 (Invitrogen) at 5 μg/mL. Because selectin binding is Ca^2+^ dependent, control experiments for all variants were performed, which included 10 mM EDTA in the binding buffer instead of CaCl_2_. Microarray slides were scanned with a Genepix 4300A (Molecular Devices) and quantified with Genepix Pro-7 software. The results are shown as relative fluorescent units by averaging the background-subtracted signals of the four replicate spots (after throwing out the highest and lowest value of the six printed spots), with error bars representing the SD of the four averaged values.

## Supplementary Material

Supplementary File

## Data Availability

All study data are included in the article and/or *SI Appendix*.

## References

[1] K. L. Moore, The biology and enzymology of protein tyrosine *O*-sulfation. J. Biol. Chem. 278, 24243–24246 (2003). 12730193 10.1074/jbc.R300008200

[2] S. M. Muthana, C. T. Campbell, J. C. Gildersleeve, Modifications of glycans: Biological significance and therapeutic opportunities. ACS Chem. Biol. 7, 31–43 (2012). 22195988 10.1021/cb2004466 PMC3262866

[3] C. I. Gama , Sulfation patterns of glycosaminoglycans encode molecular recognition and activity. Nat. Chem. Biol. 2, 467–473 (2006). 16878128 10.1038/nchembio810

[4] I. H. K. Dias , Sulfate-based lipids: Analysis of healthy human fluids and cell extracts. Chem. Phys. Lipids 221, 53–64 (2019). 30910732 10.1016/j.chemphyslip.2019.03.009

[5] K. Honke , Paranodal junction formation and spermatogenesis require sulfoglycolipids. Proc. Natl. Acad. Sci. U.S.A. 99, 4227–4232 (2002). 11917099 10.1073/pnas.032068299 PMC123630

[6] I. Fernández-Vega , Specific genes involved in synthesis and editing of heparan sulfate proteoglycans show altered expression patterns in breast cancer. BMC Cancer 13, 24 (2013). 23327652 10.1186/1471-2407-13-24 PMC3561094

[7] C. Ricciardelli , Elevated stromal chondroitin sulfate glycosaminoglycan predicts progression in early-stage prostate cancer. Clin. Cancer Res. 3, 983–992 (1997). 9815775

[8] B. Xia, J. A. Royall, G. Damera, G. P. Sachdev, R. D. Cummings, Altered *O*-glycosylation and sulfation of airway mucins associated with cystic fibrosis. Glycobiology 15, 747–775 (2005). 15994837 10.1093/glycob/cwi061

[9] G. Sundblad, S. Kajiji, V. Quaranta, H. H. Freeze, A. Varki, Sulfated *N*-linked oligosaccharides in mammalian cells. III. Characterization of a pancreatic carcinoma cell surface glycoprotein with *N*- and *O*-sulfate esters on asparagine-linked glycans. J. Biol. Chem. 263, 8897–8903 (1988). 3379052

[10] T. P. Mawhinney, E. Adelstein, D. A. Morris, A. M. Mawhinney, G. J. Barbero, Structure determination of five sulfated oligosaccharides derived from tracheobronchial mucus glycoproteins. J. Biol. Chem. 262, 2994–3001 (1987). 3029097

[11] A. H. K. Plaas, L. A. West, S. Wong-Palms, F. R. T. Nelson, Glycosaminoglycan sulfation in human osteoarthritis. Disease-related alterations at the non-reducing termini of chondroitin and dermatan sulfate. J. Biol. Chem. 273, 12642–12649 (1998). 9575226 10.1074/jbc.273.20.12642

[12] M. T. Bayliss, D. Osborne, S. Woodhouse, C. Davidson, Sulfation of chondroitin sulfate in human articular cartilage. The effect of age, topographical position, and zone of cartilage on tissue composition. J. Biol. Chem. 274, 15892–15900 (1999). 10336494 10.1074/jbc.274.22.15892

[13] M. Lei, M. V. Novotny, Y. Mechref, Sequential enrichment of sulfated glycans by strong anion-exchange chromatography prior to mass spectrometric measurements. J. Am. Soc. Mass Spectrom. 21, 348–357 (2010). 20022260 10.1016/j.jasms.2009.09.017

[14] M. Lei, Y. Mechref, M. V. Novotny, Structural analysis of sulfated glycans by sequential double-permethylation using methyl iodide and deuteromethyl iodide. J. Am. Soc. Mass Spectrom. 20, 1660–1671 (2009). 19546010 10.1016/j.jasms.2009.05.001

[15] X. Bai, J. R. Brown, A. Varki, J. D. Esko, Enhanced 3-*O*-sulfation of galactose in Asn-linked glycans and *Maackia amurensis* lectin binding in a new Chinese hamster ovary cell line. Glycobiology 11, 621–632 (2001). 11479273 10.1093/glycob/11.8.621

[16] W. C. Wang, R. D. Cummings, The immobilized leukoagglutinin from the seeds of *Maackia amurensis* binds with high affinity to complex-type Asn-linked oligosaccharides containing terminal sialic acid-linked alpha-2,3 to penultimate galactose residues. J. Biol. Chem. 263, 4576–4585 (1988). 3350806

[17] H. Tateno , Dual specificity of Langerin to sulfated and mannosylated glycans via a single C-type carbohydrate recognition domain. J. Biol. Chem. 285, 6390–6400 (2010). 20026605 10.1074/jbc.M109.041863 PMC2825434

[18] W. Gao, Y. Xu, J. Liu, M. Ho, Epitope mapping by a Wnt-blocking antibody: Evidence of the Wnt binding domain in heparan sulfate. Sci. Rep. 6, 26245 (2016). 27185050 10.1038/srep26245 PMC4869111

[19] G. B. ten Dam , Antibody GD3G7 selected against embryonic glycosaminoglycans defines chondroitin sulfate-E domains highly up-regulated in ovarian cancer and involved in vascular endothelial growth factor binding. Am. J. Pathol. 171, 1324–1333 (2007). 17717144 10.2353/ajpath.2007.070111 PMC1988881

[20] N. C. Smits , The heparan sulfate motif (GlcNS6S-IdoA2S)3, common in heparin, has a strict topography and is involved in cell behavior and disease. J. Biol. Chem. 285, 41143–41151 (2010). 20837479 10.1074/jbc.M110.153791 PMC3003412

[21] K. Ohmori , P- and E-selectins recognize sialyl 6-sulfo Lewis X, the recently identified L-selectin ligand. Biochem. Biophys. Res. Commun. 278, 90–96 (2000). 11071860 10.1006/bbrc.2000.3768

[22] R. Kannagi , Sialylated and sulfated carbohydrate ligands for selectins and siglecs: Involvement in traffic and homing of human memory T and B lymphocytes. Adv. Exp. Med. Biol. 705, 549–569 (2011). 21618129 10.1007/978-1-4419-7877-6_29

[23] J. A. O’Sullivan, D. J. Carroll, B. S. Bochner, Glycobiology of eosinophilic inflammation: Contributions of Siglecs, glycans, and other glycan-binding proteins. Front. Med. (Lausanne) 4, 116 (2017). 28824909 10.3389/fmed.2017.00116 PMC5539825

[24] M. P. Lenza, U. Atxabal, I. Oyenarte, J. Jiménez-Barbero, J. Ereño-Orbea, Current status on therapeutic molecules targeting Siglec receptors. Cells 9, 2691 (2020). 10.3390/cells9122691 PMC7765293 33333862

[25] J. Jung , Carbohydrate sulfation as a mechanism for fine-tuning Siglec ligands. ACS Chem. Biol. 16, 2673–2689 (2021). 34661385 10.1021/acschembio.1c00501

[26] C. Büll , Probing the binding specificities of human Siglecs by cell-based glycan arrays. Proc. Natl. Acad. Sci. U.S.A. 118, e2026102118 (2021). 33893239 10.1073/pnas.2026102118 PMC8092401

[27] H. Yu , Siglec-8 and Siglec-9 binding specificities and endogenous airway ligand distributions and properties. Glycobiology 27, 657–668 (2017). 28369504 10.1093/glycob/cwx026 PMC5458540

[28] B. S. Bochner , Glycan array screening reveals a candidate ligand for Siglec-8. J. Biol. Chem. 280, 4307–4312 (2005). 15563466 10.1074/jbc.M412378200

[29] R. P. Schleimer, R. L. Schnaar, B. S. Bochner, Regulation of airway inflammation by Siglec-8 and Siglec-9 sialoglycan ligand expression. Curr. Opin. Allergy Clin. Immunol. 16, 24–30 (2016). 26694037 10.1097/ACI.0000000000000234 PMC4811040

[30] J. Hirakawa , Novel anti-carbohydrate antibodies reveal the cooperative function of sulfated *N*- and *O*-glycans in lymphocyte homing. J. Biol. Chem. 285, 40864–40878 (2010). 20929857 10.1074/jbc.M110.167296 PMC3003387

[31] Z. L. Wu, B. Prather, C. M. Ethen, A. Kalyuzhny, W. Jiang, Detection of specific glycosaminoglycans and glycan epitopes by in vitro sulfation using recombinant sulfotransferases. Glycobiology 21, 625–633 (2011). 21169395 10.1093/glycob/cwq204

[32] G. Wisowski, A. Pudełko, K. Olczyk, M. Paul-Samojedny, E. M. Koźma, Dermatan sulfate affects breast cancer cell function via the induction of necroptosis. Cells 11, 173 (2022). 35011734 10.3390/cells11010173 PMC8750542

[33] T. R. McKitrick , Novel lamprey antibody recognizes terminal sulfated galactose epitopes on mammalian glycoproteins. Commun. Biol. 4, 674 (2021). 34083726 10.1038/s42003-021-02199-7 PMC8175384

[34] P. Chopra , The 3-*O*-sulfation of heparan sulfate modulates protein binding and lyase degradation. Proc. Natl. Acad. Sci. U.S.A. 118, e2012935118 (2021). 33441484 10.1073/pnas.2012935118 PMC7826381

[35] D. Pavlovic , Chemically synthesized solid phase oligosaccharide probes for carbohydrate-binding receptors. Interactions of the E-, L- and P-selectins with sialyl-Le(x) and *O*-sulphated forms linked to biotin or to polyacrylamide. J. Immunol. Methods 264, 53–58 (2002). 12191509 10.1016/s0022-1759(02)00079-0

[36] A. van Zante, S. D. Rosen, Sulphated endothelial ligands for L-selectin in lymphocyte homing and inflammation. Biochem. Soc. Trans. 31, 313–317 (2003). 12653627 10.1042/bst0310313

[37] K. Drickamer, Engineering galactose-binding activity into a C-type mannose-binding protein. Nature 360, 183–186 (1992). 1279438 10.1038/360183a0

[38] P. Adhikari , Mutational analysis at Asn-41 in peanut agglutinin. A residue critical for the binding of the tumor-associated Thomsen-Friedenreich antigen. J. Biol. Chem. 276, 40734–40739 (2001). 11447220 10.1074/jbc.M103040200

[39] D. Hu, H. Tateno, T. Sato, H. Narimatsu, J. Hirabayashi, Tailoring GalNAcα1-3Galβ-specific lectins from a multi-specific fungal galectin: Dramatic change of carbohydrate specificity by a single amino-acid substitution. Biochem. J. 453, 261–270 (2013). 23611418 10.1042/BJ20121901

[40] D. Hu, H. Tateno, A. Kuno, R. Yabe, J. Hirabayashi, Directed evolution of lectins with sugar-binding specificity for 6-sulfo-galactose. J. Biol. Chem. 287, 20313–20320 (2012). 22493425 10.1074/jbc.M112.351965 PMC3370213

[41] S. Thamotharan , Modification of the sugar specificity of a plant lectin: Structural studies on a point mutant of *Erythrina corallodendron* lectin. Acta Crystallogr. D Biol. Crystallogr. 67, 218–227 (2011). 21358053 10.1107/S0907444911004525

[42] K. Imamura, H. Takeuchi, R. Yabe, H. Tateno, J. Hirabayashi, Engineering of the glycan-binding specificity of *Agrocybe cylindracea* galectin towards α(2,3)-linked sialic acid by saturation mutagenesis. J. Biochem. 150, 545–552 (2011). 21813503 10.1093/jb/mvr094

[43] W. C. Chang , Recombinant chimeric lectins consisting of mannose-binding lectin and L-ficolin are potent inhibitors of influenza A virus compared with mannose-binding lectin. Biochem. Pharmacol. 81, 388–395 (2011). 21035429 10.1016/j.bcp.2010.10.012 PMC3053085

[44] I. C. Michelow , A novel L-ficolin/mannose-binding lectin chimeric molecule with enhanced activity against Ebola virus. J. Biol. Chem. 285, 24729–24739 (2010). 20516066 10.1074/jbc.M110.106260 PMC2915709

[45] R. Yabe , Tailoring a novel sialic acid-binding lectin from a ricin-B chain-like galactose-binding protein by natural evolution-mimicry. J. Biochem. 141, 389–399 (2007). 17234683 10.1093/jb/mvm043

[46] K. Yamamoto, I. N. Maruyama, T. Osawa, Cyborg lectins: Novel leguminous lectins with unique specificities. J. Biochem. 127, 137–142 (2000). 10731676 10.1093/oxfordjournals.jbchem.a022575

[47] K. Yamamoto, Y. Konami, T. Osawa, A chimeric lectin formed from *Bauhinia purpurea* lectin and *Lens culinaris* lectin recognizes a unique carbohydrate structure. J. Biochem. 127, 129–135 (2000). 10731675 10.1093/oxfordjournals.jbchem.a022573

[48] E. T. Jordan, I. J. Goldstein, Site-directed mutagenesis studies on the lima bean lectin. Altered carbohydrate-binding specificities result from single amino acid substitutions. Eur. J. Biochem. 230, 958–964 (1995). 7601159 10.1111/j.1432-1033.1995.tb20642.x

[49] R. Arango , Modification by site-directed mutagenesis of the specificity of *Erythrina corallodendron* lectin for galactose derivatives with bulky substituents at C-2. FEBS Lett. 330, 133–136 (1993). 8365483 10.1016/0014-5793(93)80258-v

[50] K. Yamamoto, Y. Konami, T. Osawa, T. Irimura, Alteration of the carbohydrate-binding specificity of the *Bauhinia purpurea* lectin through the preparation of a chimeric lectin. J. Biochem. 111, 87–90 (1992). 1607368 10.1093/oxfordjournals.jbchem.a123724

[51] W. S. Somers, J. Tang, G. D. Shaw, R. T. Camphausen, Insights into the molecular basis of leukocyte tethering and rolling revealed by structures of P- and E-selectin bound to SLe(X) and PSGL-1. Cell 103, 467–479 (2000). 11081633 10.1016/s0092-8674(00)00138-0

[52] M. Rösch , Synthetic inhibitors of cell adhesion: A glycopeptide from E-selectin ligand 1 (ESL-1) with the arabino sialyl Lewis(x) structure. Angew. Chem. Int. Ed. Engl. 40, 3836–3839 (2001). 10.1002/1521-3773(20011015)40:20<3836::AID-ANIE3836>3.0.CO;2-5 29712138

[53] A. Hidalgo, A. J. Peired, M. Wild, D. Vestweber, P. S. Frenette, Complete identification of E-selectin ligands on neutrophils reveals distinct functions of PSGL-1, ESL-1, and CD44. Immunity 26, 477–489 (2007). 17442598 10.1016/j.immuni.2007.03.011 PMC4080624

[54] E. L. Berg, J. Magnani, R. A. Warnock, M. K. Robinson, E. C. Butcher, Comparison of L-selectin and E-selectin ligand specificities: The L-selectin can bind the E-selectin ligands sialyl Le(x) and sialyl Le(a). Biochem. Biophys. Res. Commun. 184, 1048–1055 (1992). 1374233 10.1016/0006-291x(92)90697-j

[55] G. Weitz-Schmidt , An E-selectin binding assay based on a polyacrylamide-type glycoconjugate. Anal. Biochem. 238, 184–190 (1996). 8660609 10.1006/abio.1996.0273

[56] S. D. Rodgers, R. T. Camphausen, D. A. Hammer, Tyrosine sulfation enhances but is not required for PSGL-1 rolling adhesion on P-selectin. Biophys. J. 81, 2001–2009 (2001). 11566773 10.1016/S0006-3495(01)75850-X PMC1301674

[57] R. C. Preston , E-selectin ligand complexes adopt an extended high-affinity conformation. J. Mol. Cell Biol. 8, 62–72 (2016). 26117840 10.1093/jmcb/mjv046 PMC4710209

[58] J. M. Pröpster , Structural basis for sulfation-dependent self-glycan recognition by the human immune-inhibitory receptor Siglec-8. Proc. Natl. Acad. Sci. U.S.A. 113, E4170–E4179 (2016). 27357658 10.1073/pnas.1602214113 PMC4961179

[59] P. A. Kollman , Calculating structures and free energies of complex molecules: Combining molecular mechanics and continuum models. Acc. Chem. Res. 33, 889–897 (2000). 11123888 10.1021/ar000033j

[60] D. F. Thieker, J. A. Hadden, K. Schulten, R. J. Woods, 3D implementation of the symbol nomenclature for graphical representation of glycans. Glycobiology 26, 786–787 (2016). 27514939 10.1093/glycob/cww076 PMC5018049

[61] A. K. Nivedha, S. Makeneni, B. L. Foley, M. B. Tessier, R. J. Woods, Importance of ligand conformational energies in carbohydrate docking: Sorting the wheat from the chaff. J. Comput. Chem. 35, 526–539 (2014). 24375430 10.1002/jcc.23517 PMC3936473

[62] A. Varki , Symbol nomenclature for graphical representations of glycans. Glycobiology 25, 1323–1324 (2015). 26543186 10.1093/glycob/cwv091 PMC4643639

[63] S. Neelamegham ; SNFG Discussion Group, Updates to the symbol nomenclature for glycans guidelines. Glycobiology 29, 620–624 (2019). 31184695 10.1093/glycob/cwz045 PMC7335484

[64] T. Hou, J. Wang, Y. Li, W. Wang, Assessing the performance of the molecular mechanics/Poisson Boltzmann surface area and molecular mechanics/generalized Born surface area methods. II. The accuracy of ranking poses generated from docking. J. Comput. Chem. 32, 866–877 (2011). 20949517 10.1002/jcc.21666 PMC3043139

[65] V. R. Krishnamurthy , Glycopeptide analogues of PSGL-1 inhibit P-selectin *in vitro* and *in vivo*. Nat. Commun. 6, 6387 (2015). 25824568 10.1038/ncomms7387 PMC4423566

[66] L. Poppe, G. S. Brown, J. S. Philo, P. V. Nikrad, B. H. Shah, Conformation of sLe^x^ tetrasaccharide, free in solution and bound to E-, P-, and L-selectin. J. Am. Chem. Soc. 119, 1727–1736 (1997).

[67] A. Leppänen, S. P. White, J. Helin, R. P. McEver, R. D. Cummings, Binding of glycosulfopeptides to P-selectin requires stereospecific contributions of individual tyrosine sulfate and sugar residues. J. Biol. Chem. 275, 39569–39578 (2000). 10978329 10.1074/jbc.M005005200

[68] B. Ernst, Consortium for Functional Glycomics, Glycan Array Version PA_v41, primscreen_3459, E-selectin, 1 mg/mL. Deposited 20 August 2010.

[69] B. S. Bochner, Consortium for Functional Glycomics, Glycan Array Version PA_v5, primscreen_3917, Siglec-8, 200 μg/mL. Deposited 18 April 2011.

[70] R. D. Cummings, D. F. Smith, The selectin family of carbohydrate-binding proteins: Structure and importance of carbohydrate ligands for cell adhesion. BioEssays 14, 849–856 (1992). 1285423 10.1002/bies.950141210

[71] R. Huber , Crystal and molecular structure of human annexin V after refinement. Implications for structure, membrane binding and ion channel formation of the annexin family of proteins. J. Mol. Biol. 223, 683–704 (1992). 1311770 10.1016/0022-2836(92)90984-r

[72] B. J. Graves , Insight into E-selectin/ligand interaction from the crystal structure and mutagenesis of the lec/EGF domains. Nature 367, 532–538 (1994). 7509040 10.1038/367532a0

[73] A. Gonzalez-Gil , Sialylated keratan sulfate proteoglycans are Siglec-8 ligands in human airways. Glycobiology 28, 786–801 (2018). 29924315 10.1093/glycob/cwy057 PMC6142871

[74] W. Humphrey, A. Dalke, K. Schulten, VMD: Visual molecular dynamics. J. Mol. Graph. 14, 33–38, 27–28 (1996). 8744570 10.1016/0263-7855(96)00018-5

[75] D. A. Case , AMBER 15 (University of California, San Francisco, 2015).

[76] K. N. Kirschner , GLYCAM06: A generalizable biomolecular force field. Carbohydrates. J. Comput. Chem. 29, 622–655 (2008). 17849372 10.1002/jcc.20820 PMC4423547

[77] D. A. Case , The AMBER biomolecular simulation programs. J. Comput. Chem. 26, 1668–1688 (2005). 16200636 10.1002/jcc.20290 PMC1989667

[78] H. J. C. Berendsen, J. P. M. Postma, W. F. Vangunsteren, A. Dinola, J. R. Haak, Molecular-dynamics with coupling to an external bath. J. Chem. Phys. 81, 3684–3690 (1984).

[79] A. W. Götz , Routine microsecond molecular dynamics simulations with AMBER on GPUs. 1. Generalized Born. J. Chem. Theory Comput. 8, 1542–1555 (2012). 22582031 10.1021/ct200909j PMC3348677

[80] W. F. Vangunsteren, H. J. C. Berendsen, Algorithms for macromolecular dynamics and constraint dynamics. Mol. Phys. 34, 1311–1327 (1977).

[81] A. Onufriev, D. Bashford, D. A. Case, Modification of the generalized Born model suitable for macromolecules. J. Phys. Chem. B 104, 3712–3720 (2000).

[82] D. R. Roe, T. E. Cheatham, III, PTRAJ and CPPTRAJ: Software for processing and analysis of molecular dynamics trajectory data. J. Chem. Theory Comput. 9, 3084–3095 (2013). 26583988 10.1021/ct400341p

[83] A. Sood , Defining the specificity of carbohydrate–protein interactions by quantifying functional group contributions. J. Chem. Inf. Model. 58, 1889–1901 (2018). 30086239 10.1021/acs.jcim.8b00120 PMC6442460

[84] M. Karplus, J. N. Kushick, Method for estimating the configurational entropy of macromolecules. Macromolecules 14, 325–332 (1981).

[85] C. Gao , Unique binding specificities of proteins toward isomeric asparagine-linked glycans. Cell Chem. Biol. 26, 535–547.e4 (2019). 30745240 10.1016/j.chembiol.2019.01.002 PMC7597375

[86] O. Blixt , Printed covalent glycan array for ligand profiling of diverse glycan binding proteins. Proc. Natl. Acad. Sci. U.S.A. 101, 17033–17038 (2004). 15563589 10.1073/pnas.0407902101 PMC534418

