# Supplementary material for: Computationally guided conversion of the specificity of E-selectin to mimic that of Siglec-8
Source: Proc Natl Acad Sci U S A. 2022 Oct 3;119(41):e2117743119. doi: 10.1073/pnas.2117743119 (PMC9564326; doi:10.1073/pnas.2117743119)
Supplement: Supplementary File [file pnas.2117743119.sapp.pdf]

PNAS

Supplementary Information for

Computationally-guided Conversion of the Specificity of E-Selectin to Mimic that of Siglec-8

Xiaocong Wang*[†], Melinda S. Hanes[‡], Richard D. Cummings[‡], Robert J. Woods[†]

*Hubei Key Laboratory of Agricultural Bioinformatics, College of Informatics, Huazhong Agricultural University, Wuhan, Hubei, 430070, China

[†]Complex Carbohydrate Research Center, University of Georgia, Athens, GA 30602, USA

[‡]Department of Surgery, Beth Israel Deaconess Medical Center, Harvard Medical School, CLS 11087 - 3 Blackfan Circle, Boston, MA 02115, USA

* Robert J. Woods

Email: rwoods@ccrc.uga.edu

This PDF file includes:

Figures S1 to S12
Tables S1 to S6

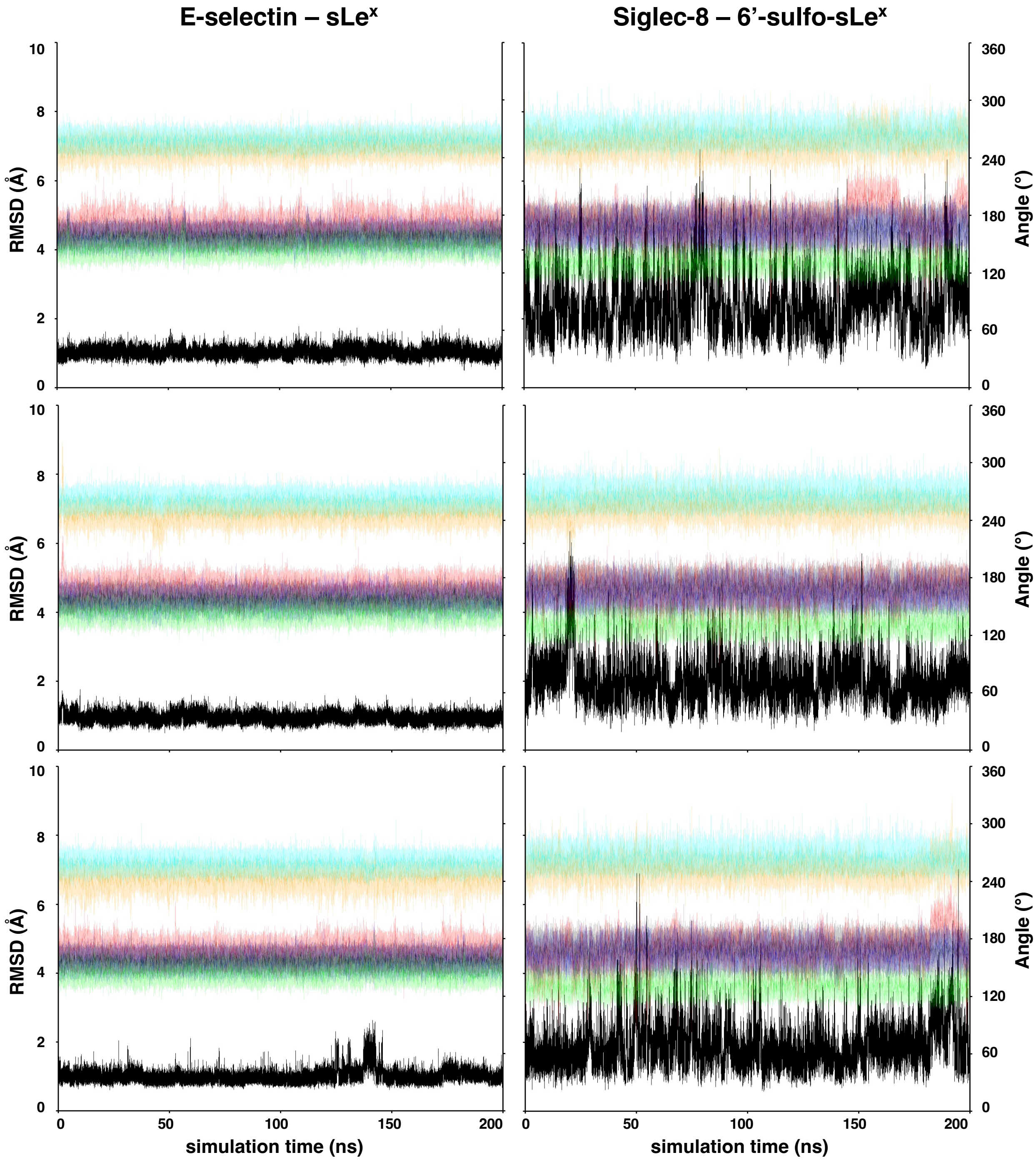

Fig. S1. Positional RMSD of ring atoms (black) and glycosidic linkages (Neu5Acα2-3Gal: φ – red, ψ – orange; Galβ1-4GlcNAc: φ – blue, ψ – green; Fucα1-3GlcNAc: φ – dark grey, ψ – cyan) in the ligands in three independent MD simulations for E-selectin – sLe^x and Siglec-8 – 6'-sulfo-sLe^x complexes. The RMSD values were computed relative to the position of the endogenous ligands in the crystal structure of the E-selectin – sLe^x and the Siglec-8 – 6'-sulfo-sLe^x complex.

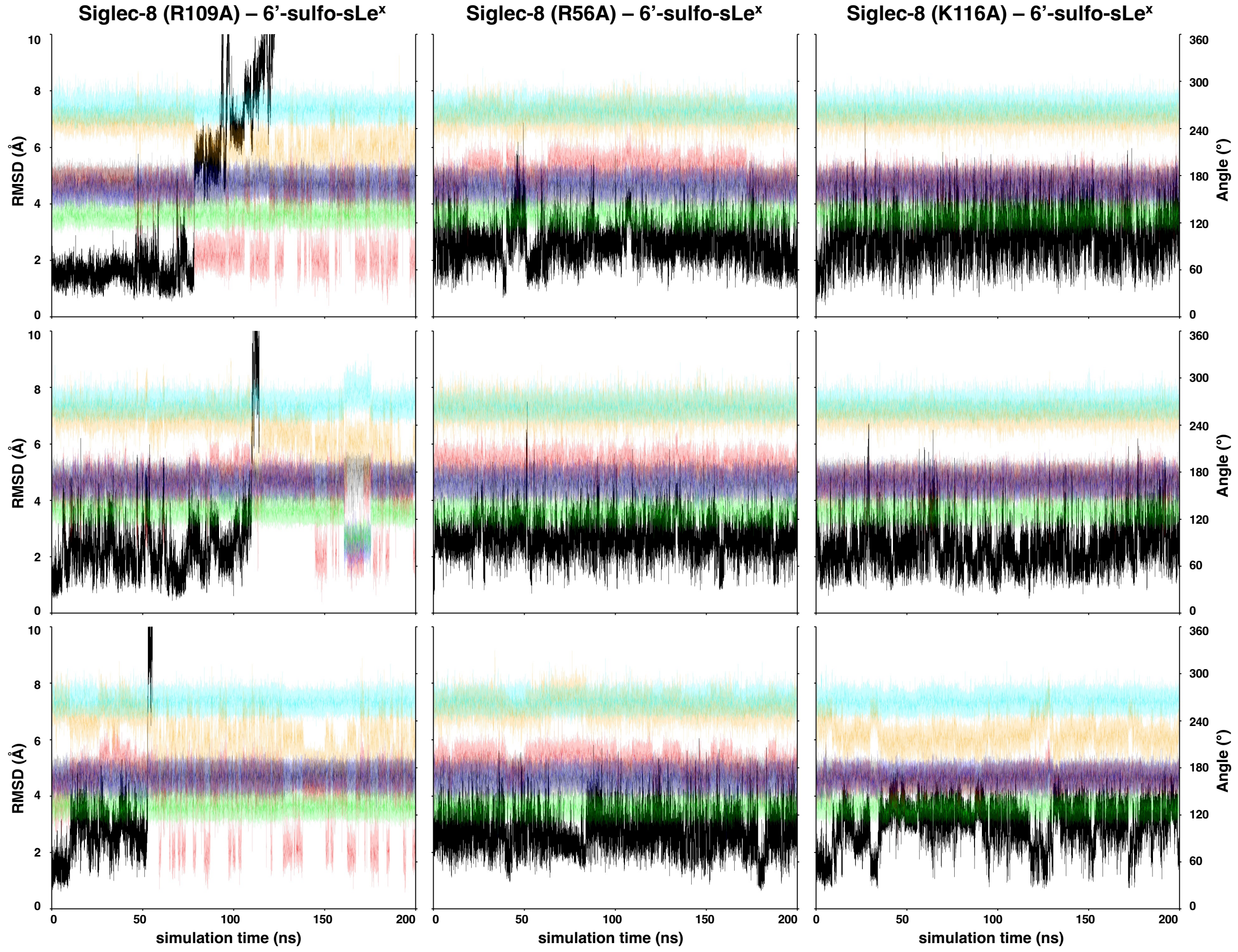

Fig. S2. Positional RMSD of ring atoms (black) and glycosidic linkages (Neu5Acα2-3Gal: φ – red, ψ – orange; Galβ1-4GlcNAc: φ – blue, ψ – green; Fucα1-3GlcNAc: φ – dark grey, ψ – cyan) in the ligands in three independent MD simulations for Siglec-8 (R109A), Siglec-8 (R56A), and Siglec-8 (K116A) complexes with 6'-sulfo-sLe^x. The RMSD values were computed relative to the position of the endogenous ligand in the crystal structure of the Siglec-8 – 6'-sulfo-sLe^x complex.

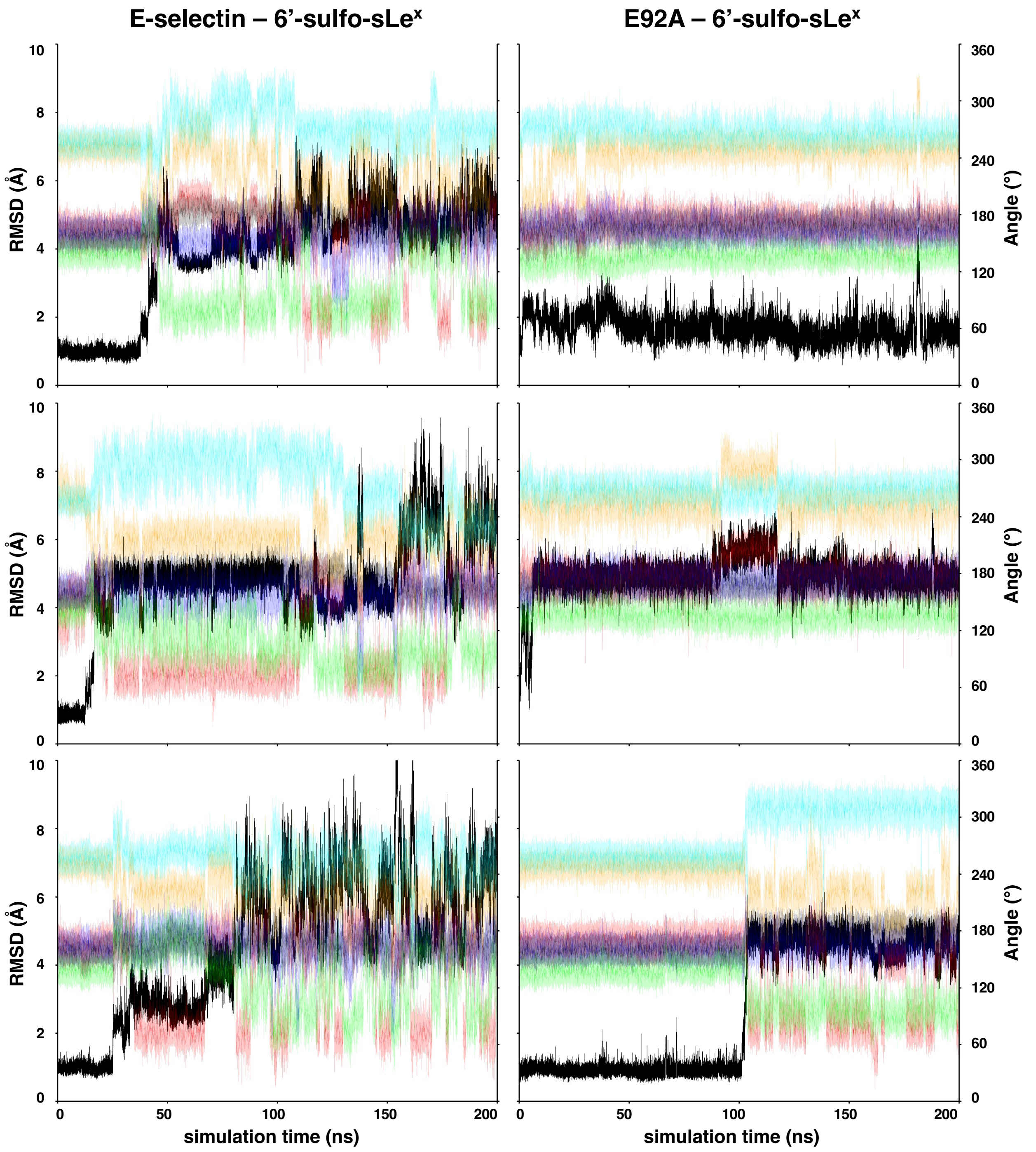

Fig. S3. Positional RMSD of ring atoms (black) and glycosidic linkages (Neu5Acα2-3Gal: φ – red, ψ – orange; Galβ1-4GlcNAc: φ – blue, ψ – green; Fucα1-3GlcNAc: φ – dark grey, ψ – cyan) in the ligands in three independent MD simulations for E-selectin – 6'-sulfo-sLe^x and E92A – 6'-sulfo-sLe^x complexes. The RMSD values were computed relative to the position of the endogenous ligand in the crystal structure of the E-selectin – sLe^x complex.

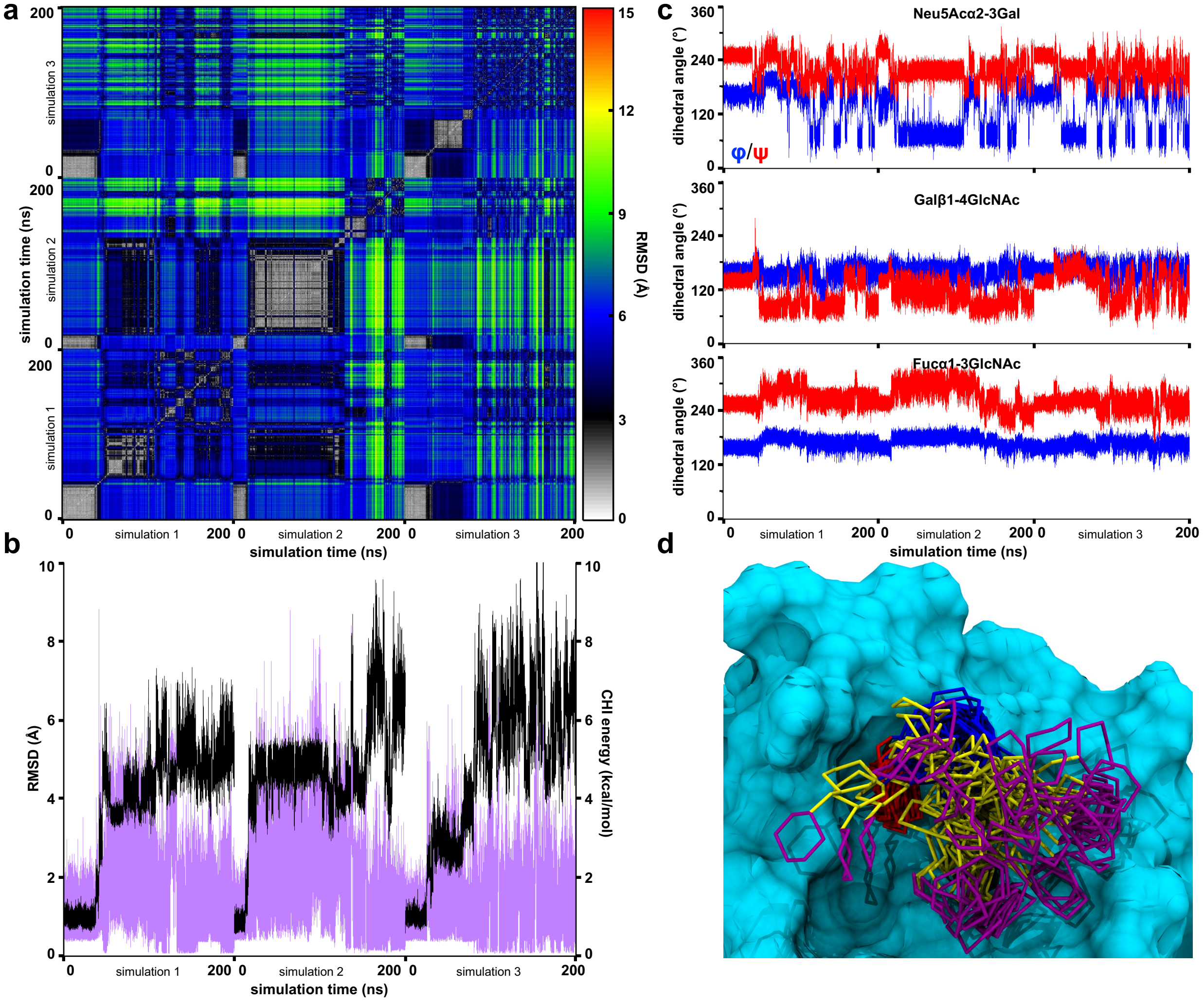

Fig. S4. Analysis of the stability of 6'-sulfo-sLe^{x} in the binding site of the wild type E-selectin. a. 2-D positional RMSD plot for the ring atoms in the ligand. Structures were evenly extracted every 0.1 ns from three MD simulations. b. Trajectories of the positional RMSD (black) for the ring atoms and the total CHI energy for the glycosidic linkages (purple) in the ligand in three MD simulations. The RMSD values were computed relative to the position of the endogenous ligand in the crystal structure of the E-selectin – sLe^{x} complex. c. Glycosidic linkages trajectories (φ: blue; ψ: red) for the ligand in three independent MD simulations; φ = C2-C1-Ox-Cx (for Neu5Ac: φ = C3-C2-Ox-Cx) and ψ = C1-Ox-Cx-Cx-1 (for Neu5Ac: ψ = C2-Ox-Cx-Cx-1). d. Superimposition of the complex for MD frames evenly extracted every 10 ns showing only the pyranose ring and glycosidic linkages atoms for clarity. The monosaccharides are colored according to SNFG nomenclature and the protein solvent accessible surface is shown in cyan.

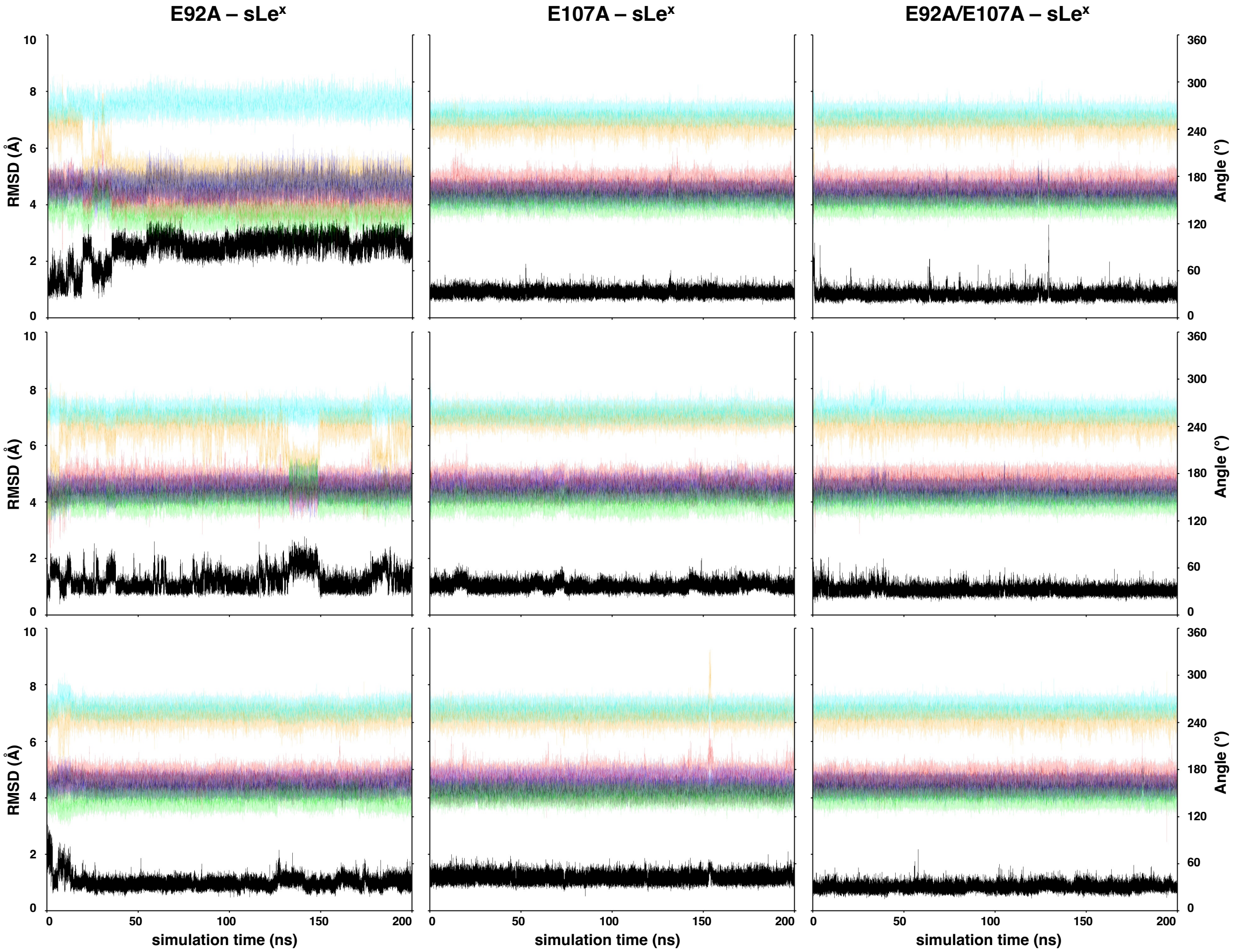

Fig. S5. Positional RMSD of ring atoms (black) and glycosidic linkages (Neu5Acα2-3Gal: φ – red, ψ – orange; Galβ1-4GlcNAc: φ – blue, ψ – green; Fucα1-3GlcNAc: φ – dark grey, ψ – cyan) in the ligands in three independent MD simulations for E92A – sLe^x, E107A – sLe^x, and E92A/E107A – sLe^x complexes. The RMSD values were computed relative to the position of the endogenous ligand in the crystal structure of the E-selectin – sLe^x complex.

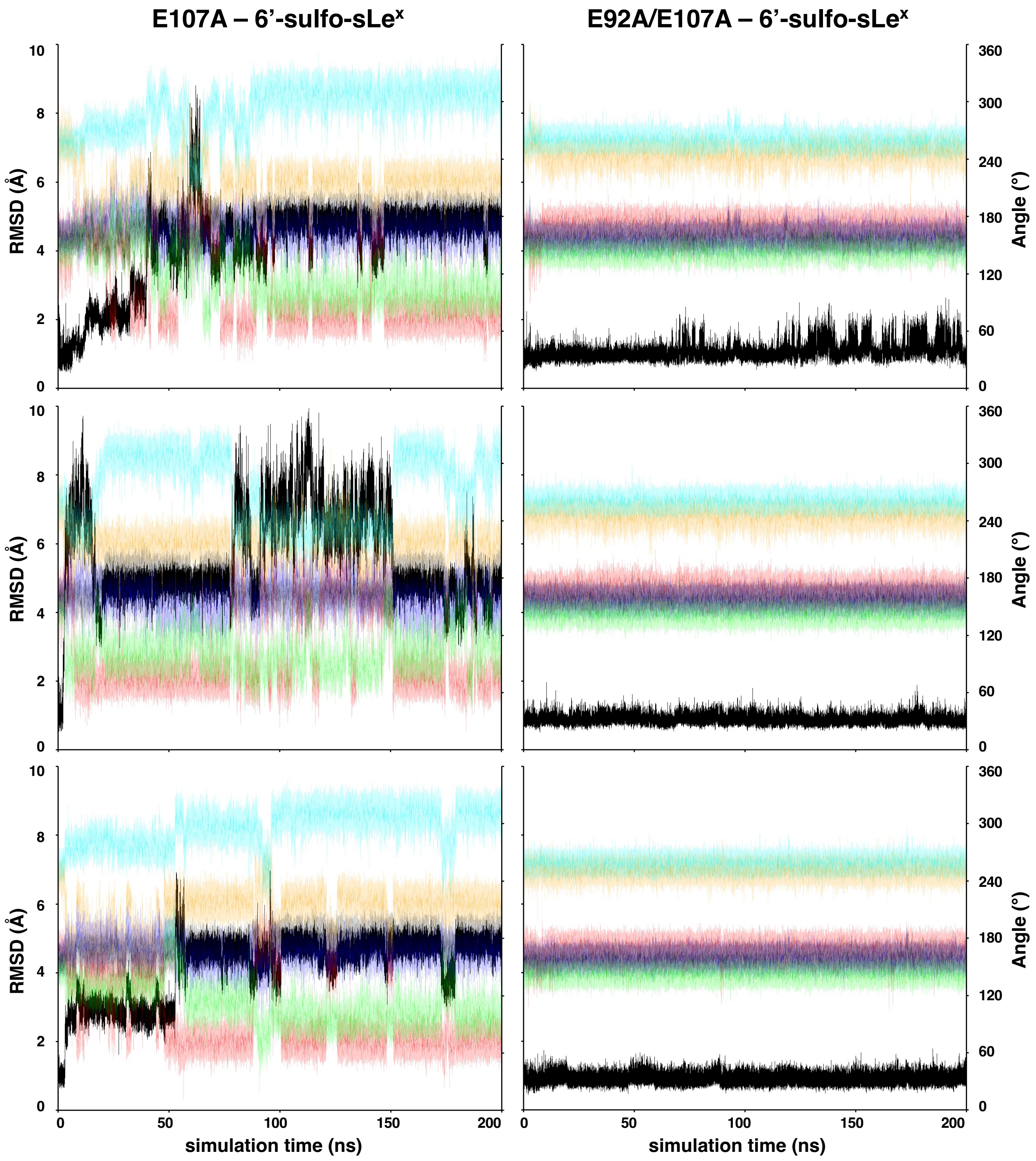

Fig. S6. Positional RMSD of ring atoms (black) and glycosidic linkages (Neu5Acα2-3Gal: φ – red, ψ – orange; Galβ1-4GlcNAc: φ – blue, ψ – green; Fucα1-3GlcNAc: φ – dark grey, ψ – cyan) in the ligands in three independent MD simulations for E107A – 6'-sulfo-sLe^x and E92A/E107A – 6'-sulfo-sLe^x complexes. The RMSD values were computed relative to the position of the endogenous ligand in the crystal structure of the E-selectin – sLe^x complex.

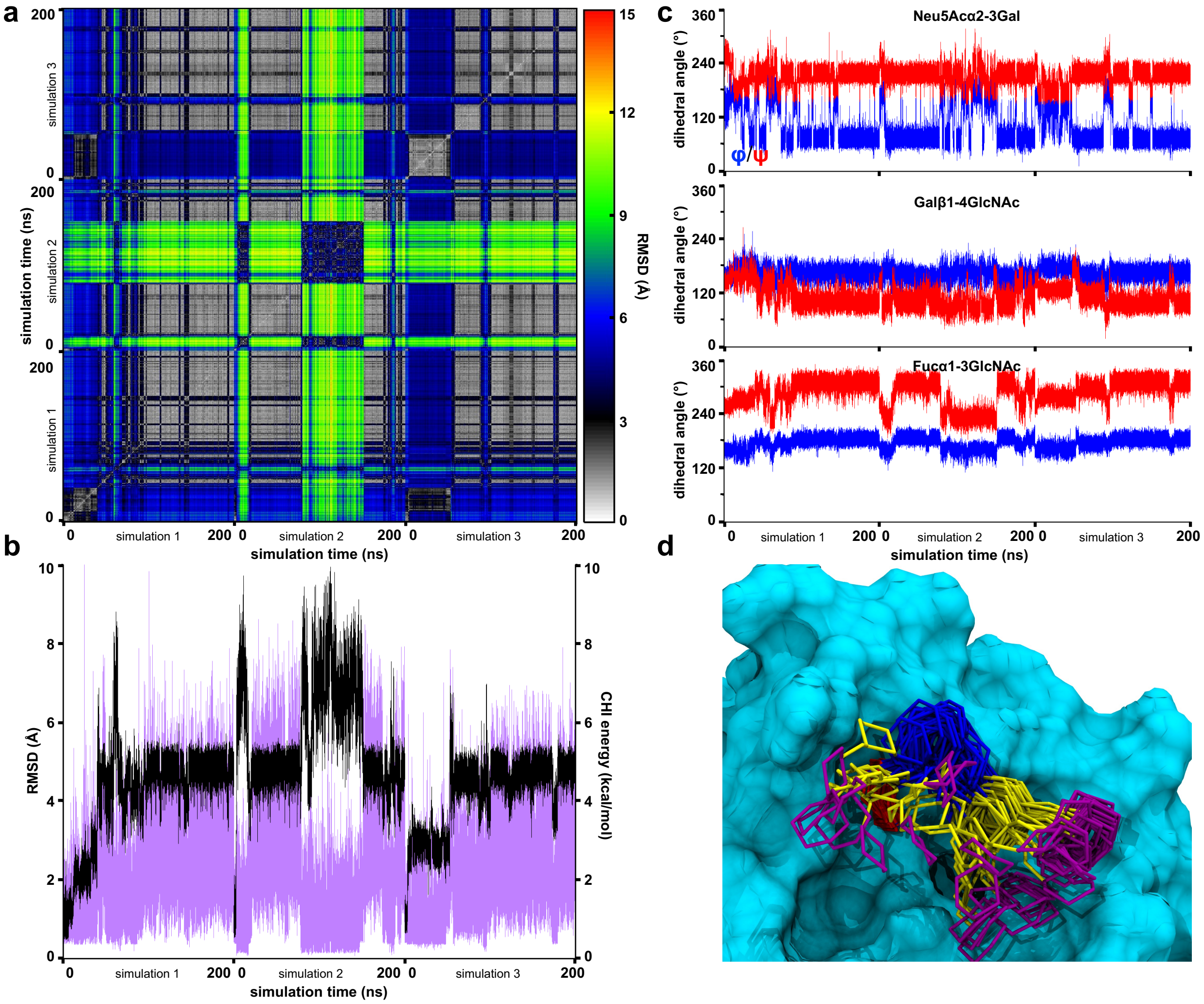

Fig. S7. Analysis of the stability of 6'-sulfo-sLe^x in the binding site of mutant E107A. a. 2-D positional RMSD plot for the ring atoms in the ligand. Structures were evenly extracted every 0.1 ns from three MD simulations and structurally related poses for the ligand are labeled (red numbers). b. Trajectories of the positional RMSD (black) for the ring atoms and the total CHI energy for the glycosidic linkages (purple) in the ligand in three MD simulations. The RMSD values were computed relative to the position of the endogenous ligand in the crystal structure of the E-selectin – sLe^x complex. c. Glycosidic linkages trajectories (φ: blue; ψ: red) for the ligand in three independent MD simulations; φ = C2-C1-Ox-Cx (for Neu5Ac: φ = C3-C2-Ox-Cx) and ψ = C1-Ox-Cx-Cx-1 (for Neu5Ac: ψ = C2-Ox-Cx-Cx-1). d. Superimposition of the complex for MD frames evenly extracted every 10 ns showing only the pyranose ring and glycosidic linkages atoms for clarity. The monosaccharides are colored according to SNFG nomenclature and the protein solvent accessible surface is shown in cyan.

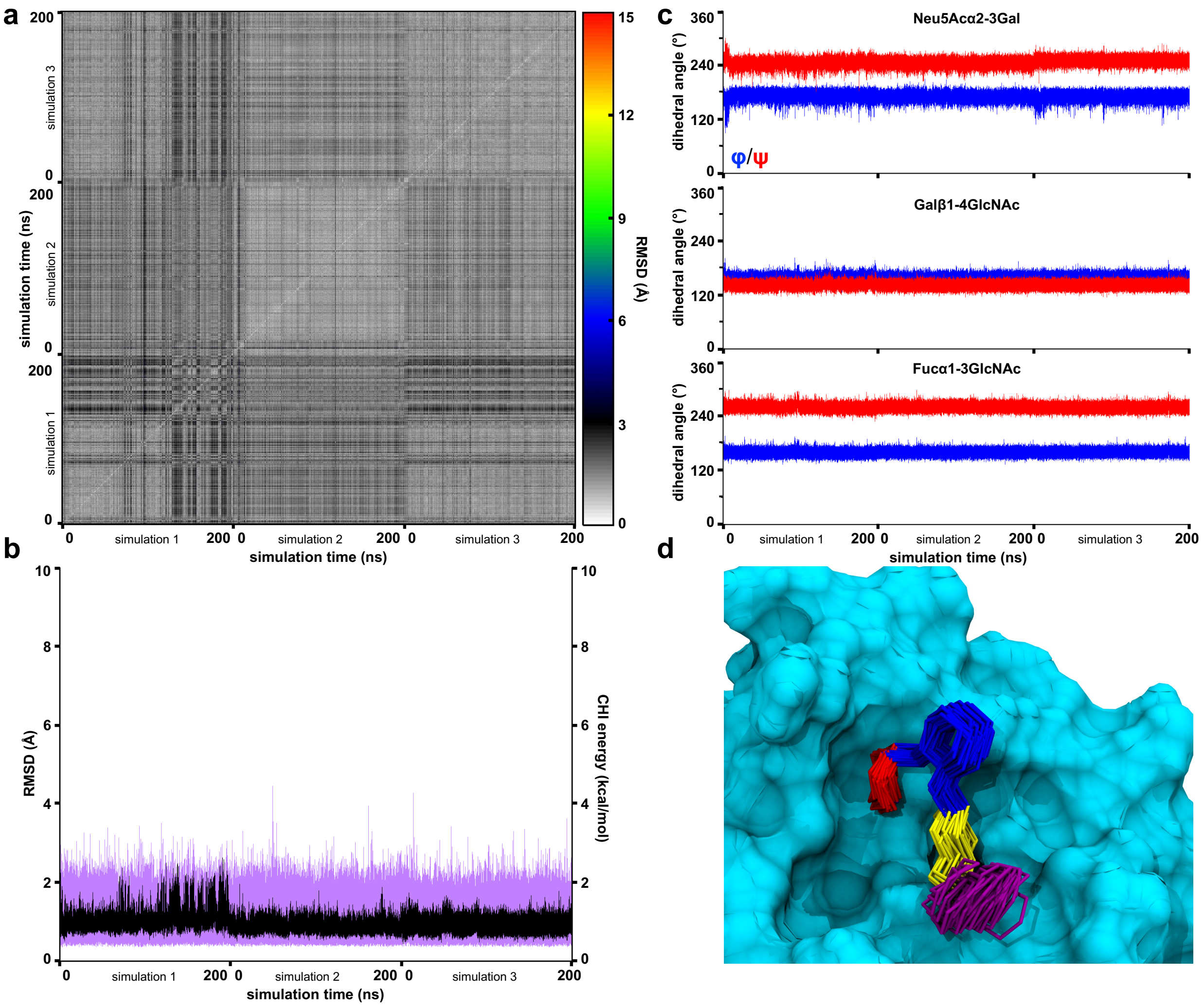

Fig. S8. Analysis of the stability of 6'-sulfo-sLe^x in the binding site of the double mutant E92A/E107A. a. 2-D positional RMSD plot for the ring atoms in the ligand. Structures were evenly extracted every 0.1 ns from three MD simulations. b. Trajectories of the positional RMSD (black) for the ring atoms and the total CHI energy for the glycosidic linkages (purple) in the ligand in three MD simulations. The RMSD values were computed relative to the position of the endogenous ligand in the crystal structure of the E-selectin – sLe^x complex. c. Glycosidic linkages trajectories (φ: blue; ψ: red) for the ligand in three independent MD simulations; φ = C2-C1-Ox-Cx (for Neu5Ac: φ = C3-C2-Ox-Cx) and ψ = C1-Ox-Cx-Cx-1 (for Neu5Ac: ψ = C2-Ox-Cx-Cx-1). d. Superimposition of the complex for MD frames evenly extracted every 10 ns showing only the pyranose ring and glycosidic linkages atoms for clarity. The monosaccharides are colored according to SNFG nomenclature and the protein solvent accessible surface is shown in cyan.

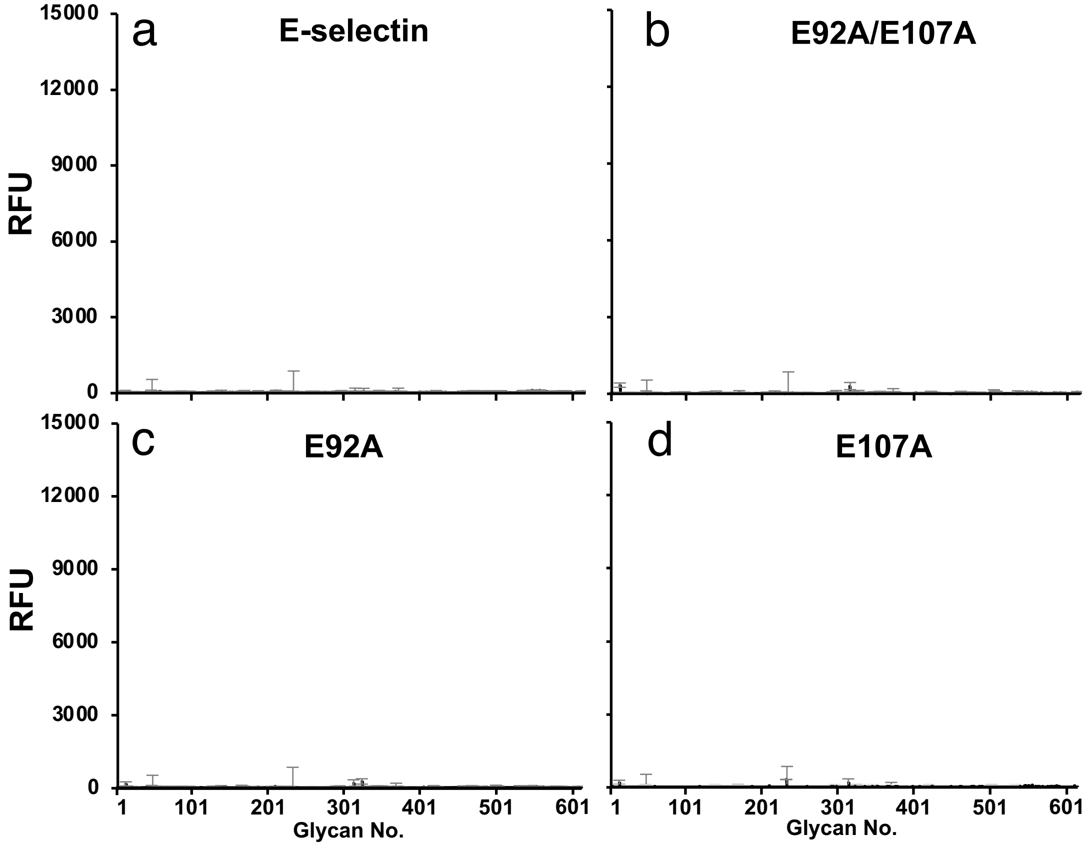

Fig. S9. Glycan microarray data measured in EDTA buffer (10 mM) for: a) wild type E-selectin, b) E92A/E107A, c) E92A, and d) E107A.

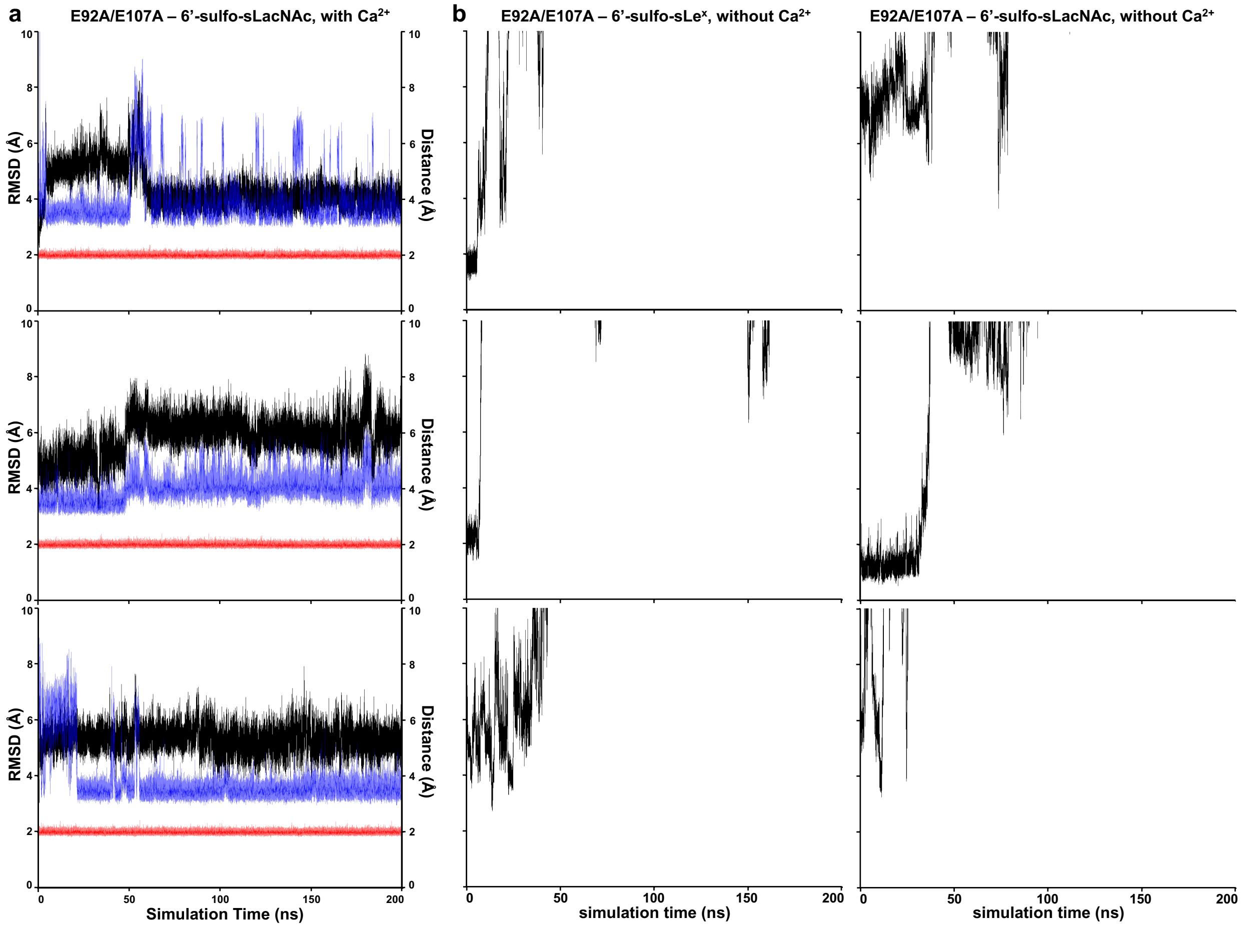

Fig. S10. a) Positional RMSD of the ring atoms (black) for 6'-sulfo-sLacNAc complexed with the E92A/E107A double mutant in the presence of the Ca^{2+} ion for three independent MD simulations (top, middle and bottom panels). The distance between the mediating water (oxygen atom) and the Ca^{2+} ion is shown in red, the distance between that water and the closest oxygen atom in the sulfate moiety in blue. b) Positional RMSD of the ring atoms (black) for 6'-sulfo-sLacNAc complexed with the E92A/E107A double mutant in the absence of the Ca^{2+} ion for three independent MD simulations (top, middle and bottom panels).

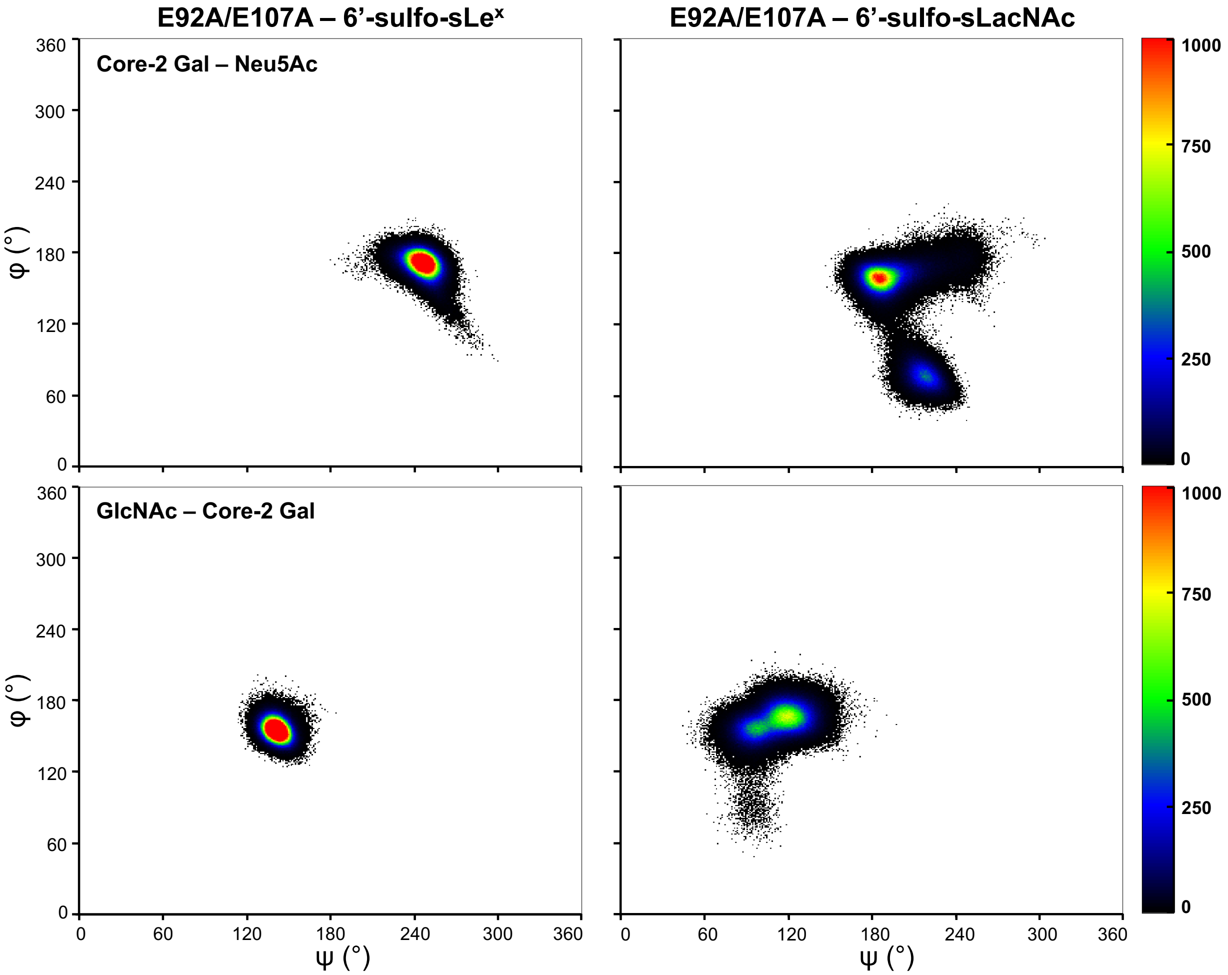

Fig. S11. The φ and ψ torsion angle distributions for the glycosidic linkages in 6’-sulfo-sLe^x (left) and 6’-sulfo-sLacNAc (right) in complexes with the double mutant for three independent MD simulations.

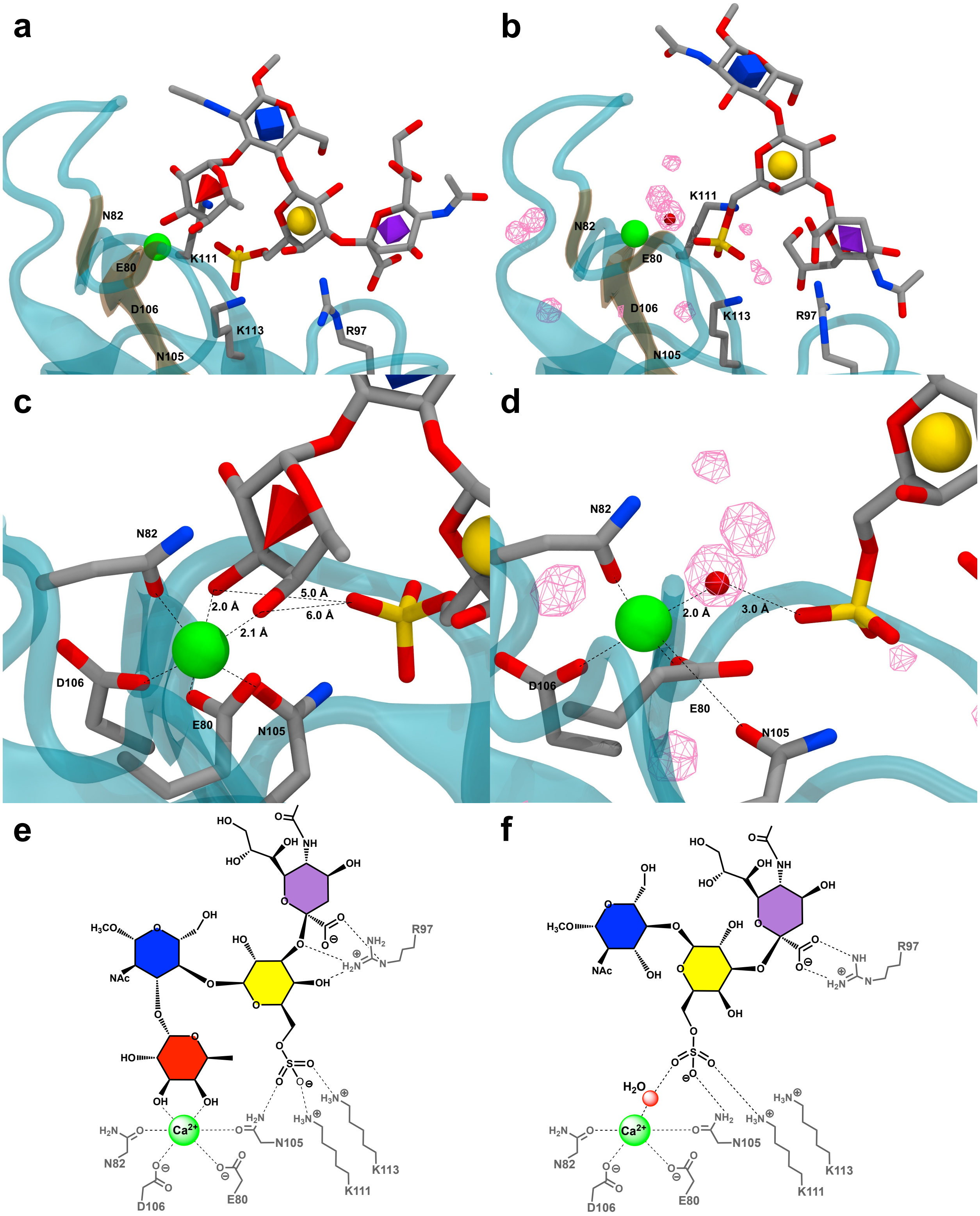

Fig. S12. Representative structures of: a) 6'-sulfo-sLe^x complexed with the double mutant. Monosaccharides are shown in 3D-SNFG icon nomenclature (Fuc, red cone; GlcNAc, blue cube;

Gal, yellow sphere; Neu5Ac, purple diamond), the Ca^{2+} ion is shown in green. Key residues that contribute to sulfate and sialic acid binding or that coordinate with the Ca^{2+} ion are labeled, with inter-atomic distances shown in following panels, b) 6'-sulfo-sLacNAc complexed with the double mutant. Areas of high occupancy for water were computed with VMD (see Methods) and are shown in pink wire frames. A water (oxygen atom) identified in the MD simulations that mediates the sulfate – Ca^{2+} ion interaction is shown as a red sphere. This water is in the equivalent position as the fucose O3 atom in the complex with 6'-sulfo-sLe^{x}, and is in the same position as that of a calcium-coordinating water in the crystal structure of the apo form of E-selectin (PDBID 1ESL), c and d) close-up views of the Ca^{2+} coordination sites in the complexes with 6'-sulfo-sLe^{x} and 6'-sulfo-sLacNAc, respectively, with key interatomic distances labeled. Note, the distance (5.0 Å) between the O3 atom of fucose and the sulfate does not support a fucose - mediated interaction with the Ca^{2+} ion in the complex with 6'-sulfo-sLe^{x}. e) and f) schematic representations of the complexes of 6'-sulfo-sLe^{x} and 6'-sulfo-sLacNAc in the double mutant, respectively, with key interactions identified.

Table S1. Essential intermolecular hydrogen bond distances and occupancies* in MD simulations of wild type and mutated E-selectin and Siglec-8 complexes.

Ligand – Atom		Protein – Atom		E-selectin – sLe^x	E92A/E107A – 6'-sulfo-sLe^x	Siglec-8 – 6'-sulfo-sLe^x
Neu5Ac	O1†	Y48	Oη	2.8 ± 0.2 (100‡)	2.8 ± 0.3 (96)	---
		R97	Nε	3.0 ± 0.2 (62)	2.9 ± 0.1 (98)	---
		R109	Nη1	---	---	3.0 ± 0.2 (79)
		R109	Nη2	---	---	2.9 ± 0.2 (100‡)
	O4	E98	Oε†	2.8 ± 0.2 (62)	2.8 ± 0.2 (63)	---
	N5	K116	O	---	---	2.9 ± 0.2 (80)
	O7	Y7	Oη	---	---	3.0 ± 0.2 (47)
	O8	R109	Nη1	---	---	3.1 ± 0.2 (52)
		Y58	Oη	---	---	3.0 ± 0.2 (49)
Gal	O3	R97	Nη2	3.0 ± 0.2 (80)	3.0 ± 0.2 (95)	---
	O4	Y94	Oη	3.0 ± 0.2 (63)	2.8 ± 0.1 (98)	---
		R97	Nη2	3.0 ± 0.1 (85)	3.1 ± 0.2 (47)	---
	O6	E92	Oε†	2.7 ± 0.1 (100‡)	---	---
Fuc	O2	E88	Oε†	2.9 ± 0.3 (100‡)	2.9 ± 0.2 (100‡)	---
	O3	E88	Oε†	2.7 ± 0.2 (100‡)	2.8 ± 0.1 (100‡)	---
		Ca^{2+}		2.0 ± 0.1 (100)	2.1 ± 0.1 (100)	---
	O4	E80	Oε†	2.6 ± 0.1 (91)	2.6 ± 0.1 (98)	---
		N82	Nδ2	3.0 ± 0.1 (72)	2.9 ± 0.1 (85)	---
		Ca^{2+}		2.1 ± 0.1 (100)	2.1 ± 0.1 (100)	---
SO_3^-	O†	N105	Nδ2	---	2.9 ± 0.2 (100‡)	---
		K111	Nζ	---	2.9 ± 0.2 (77)	---
		K113	Nζ	---	2.9 ± 0.2 (100‡)	---
		Q59	Nε2	---	---	3.0 ± 0.2 (62)
		R56	Nη2	---	---	3.0 ± 0.2 (84)
			Nε	---	---	2.9 ± 0.2 (62)

*Percentage (%) based on a distance between non-hydrogen atoms of less than 3.5Å. Distance in Å.

†when multiple hydrogen bonds are formed between multiple equivalent heavy atoms, the occupancy of the interaction listed is the sum of all the individual hydrogen bonds and the distance is the average of all the individual hydrogen bonds.

‡the occupancy of the interactions between multiple equivalent heavy atoms, calculated as the sum of all the individual hydrogen bonds, is greater than 100%.

Table S2. Per-residue MM-GBSA interaction energies* and entropies† for interactions in mutated Siglec-8 complexes.

	Siglec-8 (K116A) – 6'-sulfo-sLe^x	Siglec-8 (R56A) – 6'-sulfo-sLe^x
	**Per-residue MM-GBSA Energies**	
Neu5Ac	-12.1 ± 0.4	-12.1 ± 0.2
Core-2 Gal	-2.8 ± 0.5	-2.8 ± 0.2
GlcNAc	-1.5 ± 0.5	-2.8 ± 0.6
Fuc	-0.5 ± 0.9	-0.1 ± 0.1
SO_3 (6')	-1.3 ± 0.9	-0.2 ± 0.1
$\Delta G_{MM/GBSA}$	-18.2 ± 0.7	-18.0 ± 0.6
	**Entropic Penalties**	
$-T\Delta S_{RTV\ (all)}$	12.7 ± 1.0	14.3 ± 0.2
$-T\Delta S_q^c$	1.5 ± 0.1	1.2 ± 0.1
$-T\Delta S$	14.2 ± 0.9	15.5 ± 0.2
	**Binding free energies**	
$\Delta G_{binding}$	-4.0 ± 0.4	-2.5 ± 0.5

*in kcal/mol.

†at 300 K.

Table S3. Stable intermolecular hydrogen bond pairs for three poses* identified in the complexes of E92A – 6'-sulfo-sLe^x and E92A – sLe^x observed in the MD simulations.

		E92A – 6'-sulfo-sLe^x			E92A – sLe^x
		pose 1<br>50%†	pose 2<br>16%	pose 3<br>34%	
Neu5Ac	CO_2^-	Y48‡, R97‡	---	R97‡, K99	Y48‡, R97‡
	O4	---§	---	---	---
Core-2 Gal	O3	R97‡	---	---	R97‡
	O4	Y94‡	---	---	Y94‡
	O6	---	---	---	---
Fuc	O2	E88‡	Q85, E88‡	E107	E88‡
	O3	E107	N105	E107	E107
	O4	E80‡, N82‡	E80‡, N82‡	E80‡	E80‡, N82‡
SO_3 (6')	O	N105, K113	R97	R97	---

*poses in Figure 2.

†percentages sampled in MD simulations.

‡observed in co-crystal complex structure for E-selectin – sLe^x.

§no stable interactions observed.

Table S4. Per-residue interaction energies* and entropic penalties† for E92A with the 6'-sulfo-sLe^x and sLe^x ligands.

	E92A – 6'-sulfo-sLe^{x} ‡			E92A – sLe^{x} §
	pose1¶	pose2¶	pose3¶	
**Per-residue Interaction Energies (MM-GBSA) and Percentage of Binding**				
Neu5Ac	-2.3 (11%)	-3.0 (17%)	-2.8 (11%)	-4.4 ± 1.4 (21%)
Core-2 Gal	-3.8 (18%)	-0.4 (2%)	-3.4 (13%)	-4.1 ± 0.9 (20%)
GlcNAc	-3.4 (16%)	-5.3 (29%)	-3.7 (14%)	-3.7 ± 0.7 (17%)
Fuc	-8.8 (42%)	-8.1 (44%)	-14.6 (57%)	-8.8 ± 0.1 (42%)
SO_3 (6')	-2.6 (13%)	-1.4 (8%)	-1.2 (5%)	--
$\Delta G_{MM/GBSA}$	-20.9	-18.2	-25.7	-21.0 ± 1.2
**Entropic Penalties**				
$-T\Delta S_{RTV\ (all)}$	19.6	23.5	16.7	19.5 ± 0.4
$-T\Delta S_q^C$	1.7	-0.3	1.4	1.3 ± 0.8
$-T\Delta S$	21.3	23.2	18.1	20.8 ± 1.2
**Binding free energies**				
$\Delta G_{binding}$	0.4	5.0	-7.6	-0.2 ± 2.1

*in kcal/mol.

†at 300 K.

‡stability of the ligand in the binding site shown in Figure 2 and Figure S3.

§stability of the ligand in the binding site shown in Figure S5.

¶structures for each of the similar poses were extracted from three independent MD simulations. There were no replicas for computing error values.

Table S5. Per-residue interaction energies* and entropic penalties† for E-selectin mutants complex with sLe^x.

	E107A – sLe$^{x\ ‡}$	E92A/E107A – sLe$^{x\ ‡}$
**Per-residue Interaction Energies (MM-GBSA) and Percentage of Binding**		
Neu5Ac	-3.7 ± 0.3	-3.4 ± 0.3
Core-2 Gal	-5.5 ± 0.3	-4.1 ± 0.1
GlcNAc	-3.4 ± 0.0	-3.3 ± 0.1
Fuc	-9.6 ± 3.4	-13.3 ± 0.1
$\Delta G_{\mathrm{MM/GBSA}}$	-22.2 ± 2.8	-24.1 ± 0.5
**Entropic Penalties**		
$-\mathrm{T}\Delta S_{\mathrm{RTV\ (all)}}$	18.9 ± 1.2	19.3 ± 0.8
$-\mathrm{T}\Delta S_{\mathrm{q}}^{\mathrm{C}}$	2.3 ± 0.2	2.4 ± 0.1
$-\mathrm{T}\Delta S$	21.2 ± 1.4	21.7 ± 0.7
**Binding free energies**		
$\Delta G_{\mathrm{binding}}$	-1.0 ± 3.2	-2.4 ± 0.9

*in kcal/mol.

†at 300 K.

‡stability of the ligand in the binding site shown in Figure S5.

Table S6. Stable intermolecular hydrogen bond pairs identified in the complexes of E92A/E107A – 6’-sulfo-sLe^x and E92A/E107A – 6’-sulfo-sLacNAc observed in the MD simulations.

		E92A/E107A – 6’-sulfo-sLe^x	E92A/E107A – 6’-sulfo-sLacNAc
Neu5Ac	CO_2^-	Y48, R97	R97
	O4	E98	---
	N5	---*	Y48
	O5N	---	Y44, Y48
	O7	---	P46
	O8	---	P46
	O9	---	Y94
Core-2 Gal	O3	R97	---
	O4	Y94, R97	---
	O6	---	---
GlcNAc	O3	---	Q85, K111
Fuc	O2	E88	---
	O3	E88, Ca^{2+}	---
	O4	E80, N82, Ca^{2+}	---
SO_3 (6')	O	N105, K111, K113	N105, K111, H_2O†

*no stable interactions observed.

†water molecule that mediates the interactions between the sulfate group and Ca^{2+} ion.
